# Supplementary material for: Cancer cell adaptation to hypoxia involves a HIF‐GPRC5A‐YAP axis
Source: EMBO Mol Med. 2018 Aug 24;10(11):e8699. doi: 10.15252/emmm.201708699 (PMC6220329; doi:10.15252/emmm.201708699)
Supplement: Supplementary file 1 — Appendix [file EMMM-10-e8699-s001.pdf]

Cancer cell adaptation to hypoxia involves a HIF-GPRC5A-YAP axis

Alexander Greenhough, Clare Bagley, Kate J. Heesom, David B. Gurevich, David Gay, Mark Bond, Tracey J. Collard, Chris Paraskeva, Paul Martin, Owen J. Sansom, Karim Malik & Ann C. Williams

Appendix

Appendix Table S1
Appendix Table S2
Appendix Table S3
Appendix Table S4

Appendix Table S1.

Proteins upregulated by hypoxia >1.3-fold by SILAC proteomics

**Protein**	**Gene name [Uniprot ID]**	**Av. fold change (hyp/norm)**
Protein CIP2A OS= GN=KIAA1524 PE=1 SV=2 - [CIP2A_]	CIP2A [Q8TCG1]	9.937175
FLJ58410, to Solute carrier family 2, facilitated glucosetransporter member 3 (SLC2A3)	FLJ58410 [SLC2A3]	9.523694
FLJ58285, topre-B-cell leukemia transcription factor interacting protein 1 (PBXIP1)	FLJ58285 [PBXIP1]	6.882701
Tyrosine-protein kinase Fyn OS= GN=FYN PE=2 SV=1 - [B3KPS6_]	FYN [B3KPS6]	6.424762
Syntaxin-5 (Fragment) OS= GN=STX5 PE=4 SV=1 - [H7C3X5_]	STX5 [H7C3X5]	5.98948
FLJ76596, topreimplantation protein 3 (PREI3), transcript variant 2, mRNA OS= PE=2 SV=1 - [A8K012_]	PREI3 [A8K012]	5.845809
FLJ51483, to Multiple inositol polyphosphate phosphatase 1 (EC 3.1.3.62) OS= PE=2 SV=1 - [B4E394_]	FLJ51483 [B4E394_]	5.839956
FLJ54709, to Methylcrotonoyl-CoA carboxylase subunit alpha, mitochondrial (EC 6.4.1.4)	FLJ54709 [Q96RQ3]	5.731391
FLJ39243 fis, clone OCBBF2008283,to Protein NDRG1 OS= PE=2 SV=1 - [B3KU62_]	FLJ39243 NDRG1 [B3KU62]	5.648131
Uncharacterized protein C2orf47, mitochondrial OS= GN=C2orf47 PE=1 SV=1 - [CB047_]	C2orf47 [CB047]	5.418898
Glycolipid transfer protein (Fragment) OS= GN=GLTP PE=4 SV=1 - [H0YFS9_]	GLTP [H0YFS9]	5.227555
Transcription factor MafF OS= GN=MAFF PE=1 SV=2 - [MAFF_]	MAFF [MAFF]	5.224378
Egl nine homolog 1 OS= GN=EGLN1 PE=1 SV=1 - [EGLN1_]	EGLN1 [EGLN1]	5.128501
Tubulin-specific chaperone C OS= GN=TBCC PE=1 SV=2 - [TBCC_]	TBCC [TBCC]	4.730313
FADD protein (Fragment) OS= GN=FADD PE=2 SV=1 - [Q6LCB0_]	FADD [Q6LCB0]	4.551731
FLJ61359, to Adapter-relatedprotein complex 2 alpha- 2 subunit OS= PE=2 SV=1 - [B7Z5S9_]	FLJ61359 [B7Z5S9]	4.408787
Adducin 3 isoform a variant (Fragment) OS= PE=2 SV=1 - [Q59EK1_]	[Q59EK1]	4.124374
Pleckstrin homology domain-containing family A member 2 OS= GN=PLEKHA2 PE=4 SV=1 - [F5GZK0_]	PLEKHA2 [F5GZK0]	4.109901
Cytochrome b-c1 complex subunit 7 OS= GN=UQCRB PE=1 SV=2 - [QCR7_]	UQCRB [QCR7]	4.102806
ATP synthase mitochondrial F1 complex assembly factor 2 OS= GN=ATPAF2 PE=1 SV=1 - [ATPF2_]	ATPAF2 [ATPF2]	4.086017
Importin subunit alpha-4 OS= GN=KPNA4 PE=1 SV=1 - [IMA4_]	KPNA4 [IMA4]	4.03096
FLJ92928,to retinitis pigmentosa 2 (X-linked recessive) (RP2), mRNA OS= PE=2 SV=1 - [B2R6F5_]	FLJ92928 [B2R6F5]	3.896465
Interferon-induced transmembrane protein 2 (Fragment) OS= GN=IFITM2 PE=4 SV=1 - [H7BYV1_]	IFITM2 [H7BYV1]	3.832501
39S ribosomal protein L16, mitochondrial OS= GN=MRPL16 PE=1 SV=1 - [RM16_]	MRPL16 [RM16]	3.800119
Small intestine SPAK-like kinase OS= PE=2 SV=1 - [Q6E0B2_]	[Q6E0B2]	3.777222
DnaJ homolog subfamily C member 3 OS= GN=DNAJC3 PE=1 SV=1 - [DNJC3_]	DNAJC3 [DNJC3]	3.739799
Peptidyl-tRNA hydrolase ICT1, mitochondrial (Fragment) OS= GN=ICT1 PE=4 SV=1 - [J3KS15_]	ICT1 [J3KS15]	3.686984
Protein jagunal homolog 1 OS= GN=JAGN1 PE=1 SV=1 - [JAGN1_]	JAGN1 [JAGN1]	3.66049
ATP-dependent RNA helicase DHX29 OS= GN=DHX29 PE=1 SV=2 - [DHX29_]	DHX29 [DHX29]	3.606886
Procollagen-lysine,2-oxoglutarate 5-dioxygenase 2 OS= GN=PLOD2 PE=4 SV=1 - [E7ETU9_]	PLOD2 [E7ETU9]	3.573237
FLJ77742, tointegrin, alpha 5 (fibronectin receptor, alpha polypeptide), mRNA OS= PE=2 SV=1 - [A8K6A5_]	FLJ77742 [A8K6A5]	3.436392
Protein kinase C-binding protein 1 OS= GN=ZMYND8 PE=2 SV=1 - [B7ZM62_]	ZMYND8 [B7ZM62]	3.256561
Fructose-bisphosphate aldolase C OS= GN=ALDOC PE=1 SV=2 - [ALDOC_]	ALDOC [ALDOC]	2.795511
Lymphoid specific helicase variant9 OS= GN=HELLS PE=2 SV=1 - [Q6I7N8_]	HELLS [Q6I7N8]	2.760589
Cysteine and glycine-rich protein 1 OS= GN=CSRP1 PE=4 SV=1 - [E9PP21_]	CSRP1 [E9PP21]	2.705261
Carbonic anhydrase 9 OS= GN=CA9 PE=1 SV=2 - [CAH9_]	CA9 [CAH9]	2.595861
6-phosphofructo-2-kinase/fructose-2, 6-biphosphatase 4 splice isoform 5 OS= GN=PFKFB4 PE=2 SV=1 - [Q5XLC2_]	PFKFB4 [Q5XLC2]	2.454068
RNA demethylase ALKBH5 OS= GN=ALKBH5 PE=1 SV=2 - [ALKB5_]	ALKBH5 [ALKB5]	2.330931
FLJ16785 fis, clone NT2RI2015342,to Solute carrier family 2, facilitated glucose transporter member 1 OS= PE=2 SV=1	FLJ16785 SLC2A1 [B3KVN0]	2.312908
Coiled-coil-helix-coiled-coil-helix domain-containing protein 2, mitochondrial OS= GN=CHCHD2 PE=1 SV=1 - [CHCH2_]	CHCHD2 [CHCH2]	2.149897
[Pyruvate dehydrogenase [lipoamide]] kinase isozyme 1, mitochondrial OS= GN=PDK1 PE=2 SV=1 - [B7Z7N6_]	PDK1 [B7Z7N6]	2.147474
Metallo-beta-lactamase domain-containing protein 2 OS= GN=MBLAC2 PE=1 SV=3 - [MBLC2_]	MBLAC2 [MBLC2]	2.114094
P4HA2 protein OS= GN=P4HA2 PE=2 SV=1 - [Q05DA4_]	P4HA2 [Q05DA4]	2.082993
, FLJ96656,to SPFH domain family, member 1 (SPFH1), mRNA OS= PE=2 SV=1 - [B2RDK6_]	SPFH1 [B2RDK6]	2.036835
Cellular retinoic acid binding protein 2 (Fragment) OS= GN=CRABP2 PE=2 SV=1 - [Q5SYZ4_]	CRABP2 [Q5SYZ4]	1.942341
FLJ52712, to Tubulin beta-6 chain OS= PE=2 SV=1 - [B4DP54_]	FLJ52712 [B4DP54_]	1.927083
E-Cad/CTF3 OS= GN=CDH1 PE=4 SV=1 - [H3BVI7_]	CDH1 [H3BVI7]	1.915548
Neuroserpin OS= GN=SERPINI1 PE=3 SV=1 - [C9J6F7_]	SERPINI1 [C9J6F7]	1.859225
Prolyl 4-hydroxylase subunit alpha-1 OS= GN=P4HA1 PE=1 SV=2 - [P4HA1_]	P4HA1 [P4HA1]	1.836002
F-box/LRR-repeat protein 8 OS= GN=FBXL8 PE=1 SV=1 - [FBXL8_]	FBXL8 [FBXL8]	1.7352
Phosphoglycerate kinase 1 OS= GN=PGK1 PE=1 SV=3 - [PGK1_]	PGK1 [PGK1]	1.713839
PDZ and LIM domain protein 4 OS= GN=PDLIM4 PE=1 SV=2 - [PDLI4_]	PDLIM4 [PDLI4]	1.694211
MUC1 isoform M11 OS= GN=MUC1 PE=2 SV=1 - [A6ZIE5_]	MUC1 [A6ZIE5]	1.66769
Iron-responsive element binding protein 2, isoform CRA_a OS= GN=IREB2 PE=4 SV=1 - [D3DW85_]	IREB2 [D3DW85]	1.651273
[Pyruvate dehydrogenase [lipoamide]] kinase isozyme 3, mitochondrial OS= GN=PDK3 PE=1 SV=1 - [PDK3_]	PDK3 [PDK3]	1.648721
Protein FAM162A OS= GN=FAM162A PE=4 SV=1 - [F8W7Q4_]	FAM162A [F8W7Q4]	1.643937
2-methoxy-6-polyprenyl-1,4-benzoquinol methylase, mitochondrial (Fragment) OS= GN=COQ5 PE=4 SV=1 - [F8VVX6_]	COQ5 [F8VVX6]	1.64317
Keratin, type II cytoskeletal 80 OS= GN=KRT80 PE=1 SV=2 - [K2C80_]	KRT80 [K2C80]	1.631964
Thymidylate synthase OS= GN=TS PE=2 SV=1 - [Q8WYK3_]	TS [Q8WYK3]	1.619454
Retinoic acid-induced protein 3 (Fragment) OS= GN=GPRC5A PE=4 SV=1 - [F5GWG3_]	GPRC5A [F5GWG3]	1.617928
BRI3-binding protein OS= GN=BRI3BP PE=1 SV=1 - [BRI3B_]	BRI3BP [BRI3B]	1.564127
EF-hand domain-containing protein D2 OS= GN=EFHD2 PE=1 SV=1 - [EFHD2_]	EFHD2 [EFHD2]	1.544446
Ribonucleoside-diphosphate reductase subunit M2 (Fragment) OS= GN=RRM2 PE=4 SV=1 - [C9JXC1_]	RRM2 [C9JXC1]	1.535526
JARID1C protein OS= GN=JARID1C PE=2 SV=1 - [A6N6J7_]	JARID1C [A6N6J7]	1.528685
Ephrin-B1 OS= GN=EFNB1 PE=1 SV=1 - [EFNB1_]	EFNB1 [EFNB1]	1.515968
Hexokinase-2 OS= GN=HK2 PE=3 SV=1 - [E9PB90_]	HK2 [E9PB90]	1.509546
Protein S100-A10 OS= GN=S100A10 PE=1 SV=2 - [S10AA_]	S100A10 [S10AA]	1.505843
Methylsterol monooxygenase 1 OS= GN=MSMO1 PE=1 SV=1 - [MSMO1_]	MSMO1 [MSMO1]	1.488818
Adenylate kinase isoenzyme 4, mitochondrial OS= GN=AK4 PE=1 SV=1 - [KAD4_]	AK4 [KAD4]	1.476758
Dolichol-phosphate mannosyltransferase subunit 3 OS= GN=DPM3 PE=1 SV=2 - [DPM3_]	DPM3 [DPM3]	1.466608
FLJ53831, to FK506-binding protein 9 (EC 5.2.1.8) OS= PE=2 SV=1 - [B7Z1U8_]	FLJ53831 [B7Z1U8]	1.461006
FLJ76881, toglycogen synthase 1 (muscle) (GYS1), mRNA OS= PE=2 SV=1 - [A8K6K7_]	FLJ76881 GYS1 [A8K6K7]	1.457569
Acyl-coenzyme A oxidase OS= PE=2 SV=1 - [B4DU07_]	Acyl-coenzyme A oxidase [B4DU07]	1.450854
Cytosolic 5'-nucleotidase 3 OS= GN=NT5C3 PE=1 SV=3 - [5NT3_]	NT5C3 [5NT3]	1.433691
Enolase OS= GN=ENO2 PE=2 SV=1 - [B7Z2X9_]	ENO2 [B7Z2X9]	1.432793
Putative Golgi pH regulator C (Fragment) OS= GN=GPR89C PE=4 SV=1 - [H0YFP4_]	GPR89C [H0YFP4]	1.421682
Leucine-rich repeat and death domain-containing protein 1 OS= GN=LRRD1 PE=2 SV=2 - [LRRD1_]	LRRD1 [LRRD1]	1.414377
Chromosome 6 open reading frame 150 OS= GN=C6orf150 PE=4 SV=1 - [A6PW79_]	C6orf150 [A6PW79_]	1.409133
Retinoic acid early transcript 1H (Fragment) OS= GN=RAET1H PE=4 SV=1 - [J7HBC9_]	RAET1H [J7HBC9]	1.40526
Keratin, type I cytoskeletal 19 OS= GN=KRT19 PE=1 SV=4 - [K1C19_]	KRT19 [K1C19]	1.40006
Fructose-bisphosphate aldolase A OS= GN=ALDOA PE=1 SV=2 - [ALDOA_]	ALDOA [ALDOA]	1.384885
Niban-like protein 1 OS= GN=FAM129B PE=1 SV=3 - [NIBL1_]	FAM129B [NIBL1]	1.376295
ERO1-like protein alpha OS= GN=ERO1L PE=1 SV=2 - [ERO1A_]	ERO1L [ERO1A]	1.368026
Similar to cytoskeleton-associated protein 4 (Fragment) OS= PE=2 SV=1 - [Q8TB01_]	[Q8TB01]	1.36028
Acyl-coenzyme A oxidase OS= PE=2 SV=1 - [B2R856_]	[B2R856]	1.359386
N-chimaerin (Fragment) OS= GN=CHN1 PE=4 SV=1 - [C9J3G1_]	CHN1 [C9J3G1]	1.341558
Monocarboxylate transporter 4 OS= GN=SLC16A3 PE=1 SV=1 - [MOT4_]	SLC16A3 [MOT4]	1.334075
Peroxisomal membrane protein PMP34 OS= GN=SLC25A17 PE=3 SV=1 - [B0QYW5_]	SLC25A17 [B0QYW5]	1.327424
AGPAT2 protein (Fragment) OS= GN=AGPAT2 PE=2 SV=1 - [Q96IS9_]	AGPAT2 [Q96IS9]	1.326865
L-lactate dehydrogenase A chain OS= GN=LDHA PE=1 SV=2 - [LDHA_]	LDHA [LDHA]	1.311662
Serine/arginine-rich splicing factor 9 OS= GN=SRSF9 PE=1 SV=1 - [SRSF9_]	SRSF9 [SRSF9]	1.306569
Heterochromatin protein 1-binding protein 3 OS= GN=HP1BP3 PE=1 SV=1 - [HP1B3_]	HP1BP3 [HP1B3]	1.302614
GDH/6PGL endoplasmic bifunctional protein OS= GN=H6PD PE=1 SV=2 - [G6PE_]	H6PD [G6PE]	1.302455
FLJ39235 fis, clone OCBBF2007829,to Mus musculus fatso protein OS= PE=2 SV=1 - [B3KU60_]	FLJ39235 [B3KU60]	1.300593

Appendix Table S2.

R2: KEGG Pathway analysis
Using dataset ps_avgpres_gse24551geot333_huex10t
tumor_stage-3 140 of 333 samples, transform_2log, PresCalls>=1
Sourcegene=GPRC5A(3405587)

Group	In_Set	Total	%	p-value
Hippo_signaling_pathway	92	123	74.80%	1.10E-04
Endocytosis	131	184	71.20%	1.90E-04
Adherens_junction	50	62	80.60%	2.40E-04
Hepatitis_B	80	108	74.10%	5.30E-04
Ubiquitin_mediated_proteolysis	76	102	74.50%	5.50E-04
Cell_cycle	74	99	74.70%	5.60E-04
Lysine_degradation	27	31	87.10%	8.90E-04
Aminoacyl_tRNA_biosynthesis	24	27	88.90%	1.00E-03
Fatty_acid_degradation	23	26	88.50%	1.50E-03
Influenza_A	86	120	71.70%	1.80E-03
Herpes_simplex_infection	93	132	70.50%	2.80E-03
Glycosylphosphatidylinositol_GPI__anchor_biosynthesis	15	16	93.80%	3.40E-03
Protein_processing_in_endoplasmic_reticulum	90	128	70.30%	3.60E-03
Viral_carcinogenesis	98	141	69.50%	4.20E-03
p53_signaling_pathway	44	58	75.90%	4.90E-03
Sphingolipid_signaling_pathway	70	99	70.70%	8.30E-03
Tuberculosis	87	126	69.00%	9.30E-03
TNF_signaling_pathway	64	90	71.10%	9.50E-03
FoxO_signaling_pathway	74	106	69.80%	0.01
Bladder_cancer	24	30	80.00%	0.01
TGF_beta_signaling_pathway	49	68	72.10%	0.02
MicroRNAs_in_cancer	84	123	68.30%	0.02
Proteoglycans_in_cancer	109	163	66.90%	0.02
Apoptosis	50	70	71.40%	0.02
Sphingolipid_metabolism	27	35	77.10%	0.02
Measles	69	100	69.00%	0.02
Oocyte_meiosis	60	86	69.80%	0.02
Endometrial_cancer	28	37	75.70%	0.03
Colorectal_cancer	37	51	72.50%	0.03
Glycerolipid_metabolism	37	51	72.50%	0.03
Leishmaniasis	37	51	72.50%	0.03
AMPK_signaling_pathway	68	100	68.00%	0.04
Chronic_myeloid_leukemia	38	53	71.70%	0.04
Epstein_Barr_virus_infection	97	147	66.00%	0.04
Small_cell_lung_cancer	52	75	69.30%	0.04
mRNA_surveillance_pathway	43	61	70.50%	0.04
Lysosome	60	88	68.20%	0.04

R2: Gene Set Analysis
Tumor Colon (Core-Transcript) - Sveen - 333 - rma_sketch - huex10t
tumor_stage-3 140 of 333 samples, transform_2log, PresCalls>=1
Sourcegene=GPRC5A(3405587) within Hippo_signaling_pathway

HUGO (Gene symbol)	r value	p value	Pres
CDH1	0.57	2.00E-13	140
PARD6B	0.518	5.70E-11	140
PPP2R1B	0.513	9.10E-11	140
YAP1	0.5	3.30E-10	140
CCND1	0.495	4.90E-10	140
YWHAB	0.481	1.70E-09	140
TP53BP2	0.481	1.80E-09	140
TGFBR2	0.479	2.20E-09	140
TEAD1	0.468	5.60E-09	140
FBXW11	0.458	1.30E-08	140
BMP8A	0.451	2.20E-08	134
TCF7L2	0.449	2.70E-08	140
WNT5A	0.422	2.10E-07	136
AREG	0.42	2.40E-07	140
DLG3	0.417	3.00E-07	140
MOB1A	0.401	9.40E-07	140
PPP2R2A	0.398	1.10E-06	140
YWHAE	0.394	1.50E-06	140
YWHAZ	0.39	2.00E-06	140
PPP2R1A	0.389	2.10E-06	140
LATS1	0.388	2.20E-06	140
CTNNB1	0.386	2.50E-06	140
BMP4	0.381	3.30E-06	140
ACTG1	0.377	4.40E-06	140
PPP1CA	0.366	8.60E-06	140
MPP5	0.364	9.60E-06	140
SERPINE1	0.36	1.30E-05	140
TEAD4	0.358	1.40E-05	140
FZD6	0.356	1.60E-05	140
MYC	0.351	2.20E-05	140
YWHAG	0.334	5.50E-05	140
LATS2	0.331	6.60E-05	140
SNAI2	0.327	8.20E-05	140
TEAD3	0.324	9.50E-05	140
BMP5	0.316	1.40E-04	110
MOB1B	0.314	1.50E-04	140
PPP2CB	0.313	1.70E-04	140
INADL	0.311	1.80E-04	140
BMP2	0.311	1.80E-04	112
CCND3	0.308	2.10E-04	140
LLGL2	0.307	2.30E-04	140
AJUBA	0.306	2.40E-04	140
WWC1	0.299	3.30E-04	140
BIRC2	0.298	3.40E-04	140
FZD5	0.297	3.60E-04	140
STK3	0.297	3.60E-04	140
BMPR2	0.296	3.80E-04	140
RASSF6	0.284	6.70E-04	140
PPP1CC	0.283	6.90E-04	140
SMAD2	0.279	8.30E-04	140
ACTB	0.278	8.80E-04	140
NF2	0.277	9.40E-04	140
AXIN1	0.272	1.20E-03	140
YWHAQ	0.268	1.30E-03	140
ID1	0.268	1.40E-03	136
BTRC	0.249	3.00E-03	140
BMP7	0.241	4.10E-03	118
CTGF	0.239	4.50E-03	140
TGFBR1	0.238	4.70E-03	140
TCF7	0.235	5.30E-03	140
BMPR1A	0.231	6.00E-03	140
WNT2	0.216	0.01	132
DVL3	0.216	0.01	140
BMP6	0.213	0.01	130
DLG1	0.211	0.01	140
PRKCZ	0.196	0.02	140
APC	0.195	0.02	140
TGFB2	0.178	0.04	140
WNT11	0.177	0.04	116
TCF7L1	0.174	0.04	140
BIRC5	0.169	0.05	140
AMOT	0.166	0.05	140

Appendix Table S3. List of siRNAs used in this study

Negative control	ON-TARGETplus Non-targeting siRNA pool	D-001810-10	Dharmacon
HIF-1α siRNA 1	ON-TARGETplus HIF1A siRNA	J-004018-08	Dharmacon
HIF-1α siRNA 2	ON-TARGETplus HIF1A siRNA	J-004018-10	Dharmacon
HIF-1β	SMARTpool: ON-TARGETplus HIF1B siRNA	L-007207-00	Dharmacon
YAP1 siRNA	SMARTpool: ON-TARGETplus YAP1 siRNA	L-012200-00	
GPRC5A siRNA pool (SP)	SMARTpool: ON-TARGETplus GPRC5A siRNA	L-004576-00	Dharmacon
GPRC5A siRNA 1	ON-TARGETplus GPRC5A siRNA	J-004576-08	Dharmacon
GPRC5A siRNA 2	ON-TARGETplus GPRC5A siRNA	J-004576-07	Dharmacon
GPRC5A siRNA 3	ON-TARGETplus GPRC5A siRNA	J-004576-06	Dharmacon
HIF-2α siRNA 1	FlexiTube siRNA EPAS1_2	S100380212	Qiagen
HIF-2α siRNA 2	FlexiTube siRNA EPAS1_4	S100380226	Qiagen
Negative control	Negative control RNA Duplex	1027310	Qiagen

Appendix Table S4. List of primers used in this study

cDNA primers

**Human**	**Fwd primer 5'-3'**	**Rev primer 5'-3'**
GPRC5A	Hs_GPRC5A_2_SG QuantiTect Primer Assay	QT01153488
CYR61	ACCGCTCTGAAGGGGATCT	ACTGATGTTTACAGTTGGGCTG
AREG	GTCGATGATTCAGTCAGAGTTGA	TTCAGTTATGCTATAGCATGTACAT
CTGF	CAGCATGGACGTTCGTCTG	AACCACGGTTTGGTCCTTGG
BCL2L1	CCCTTCAGAATCTTATCTTG	TGTAGGAGAGAAAGTCAACC

**Mouse**	**Fwd primer 5'-3'**	**Rev primer 5'-3'**
Gprc5a	Mm_Rai3_1_SG QuantiTect Primer Assay	QT00104832
Car9	GGTTAGAGGATCTATCGACTCCC	GGTGCCTCCATAGCTCCAA
Egln3	AGGCAATGGTGGCTTGCTATC	GCGTCCCAATTCTTATTCAGGT
Dll4	TTCCAGGCAACCTTCTCCGA	ACTGCCGCTATTCTTGTCCC

**Zebrafish**	**Fwd primer 5'-3'**	**Rev primer 5'-3'**
gprc5ba	CAGTTCGTGGACAACCAGAG	AGAGCTGTCTGCCGGTGTAT
gprc5bb	GAGAGTCAGGCGTACACTGT	GCAGAGCCATTTCAGTAGGC
gprc5c	CCAAGCAGAGGGTACGAGAA	GCCGTTATAGCCGCTGTATG
cahz	TGAAGGAGCCGATCAGTGTT	CCATGCAGCAAGGTGTTTCT
p4ha1	CGTGATGCTCAGAGGAAACG	AAACAGGCGGCTTTGTCTAC
plod1	ACGAGGTCGTGCTGAAGTTT	TGCAGCCAGTCTCAAATGTC
ef1a	CTTCTCAGGCTGACTGTGC	CCGCTAGCATTACCCTCC

ChIP primers

**Human**	**Fwd primer 5'-3'**	**Rev primer 5'-3'**
CA9	TCTCGTTTCCAATGCACGTACAGC	AGTGACAGCAGCAGTTGCACAGT
GPRC5A	ACCCAAACACTTCCTTTGCC	AAGGCTCTGAACAAGGGAGT
GPRC5A 8	GCTGAGCTAACTACGGACCT	AGCCTTTCTCTCCATCACTCT

Cloning primers (GPRC5A si1R, AvrII and BsrgI)

**Human**	**Fwd primer 5'-3'**	**Rev primer 5'-3'**
GPRC5A si1R	AAAACCTAGGATGGCTACAACAGTCCCTGATG	AAAATGTACATTAGCTGCCCTCTTTCTTTACTTCA
