# Supplementary material for: Cancer cell adaptation to hypoxia involves a HIF‐GPRC5A‐YAP axis
Source: EMBO Mol Med. 2018 Aug 24;10(11):e8699. doi: 10.15252/emmm.201708699 (PMC6220329; doi:10.15252/emmm.201708699)
Supplement: Supplementary file 2 — Expanded View Figures PDF [file EMMM-10-e8699-s002.pdf]

Expanded View Figures

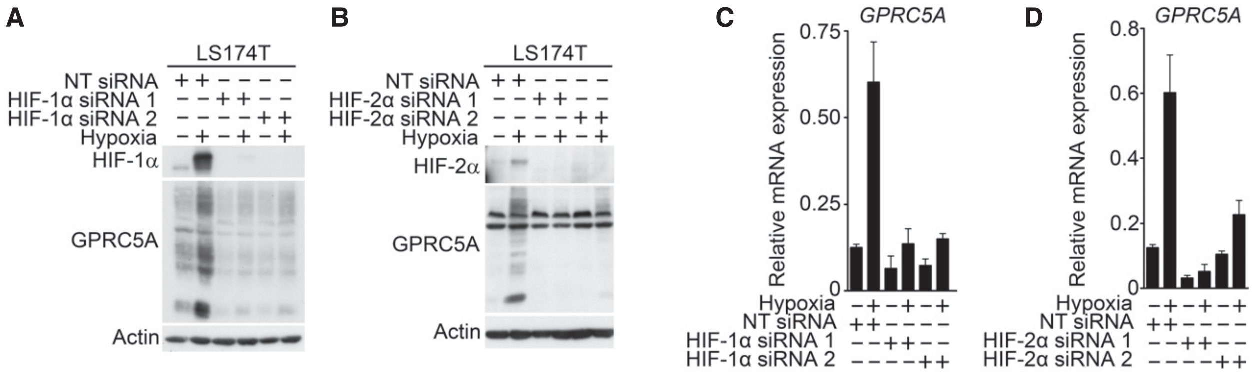

Figure EV1. GPRC5A upregulation by hypoxia requires HIFs.

A–D Knockdown of HIF-1α or HIF-2α using independent siRNAs decreased GPRC5A protein (A, B) and mRNA (C, D) upregulation by hypoxia in LS174T cells. Related to Fig 1F and J. For (C & D) representative examples of $n = 3$ independent experiments are shown; data are presented as mean ± SD.

Source data are available online for this figure.

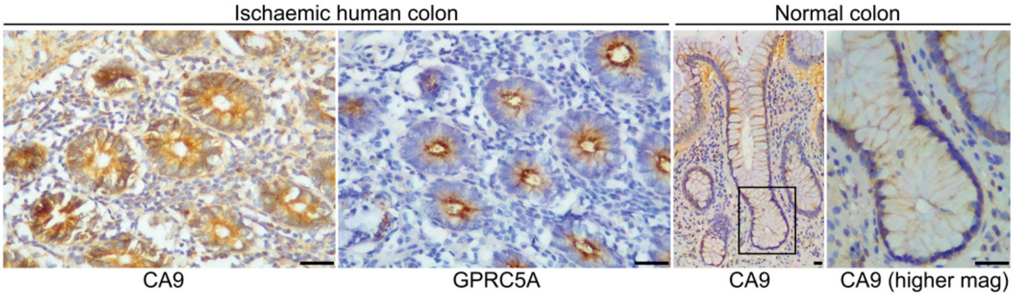

Figure EV2. GPRC5A and CA9 are expressed in mesenteric ischaemia (strangulated colon).

GPRC5A and CA9 immunohistochemistry from transverse sections of human colon from patients with mesenteric ischaemia (left panels) and CA9 immunohistochemistry from longitudinal sections of normal human colon. IHC staining confirmed high levels of CA9 and GPRC5A protein expression in hypoxic tissue (mesenteric ischaemia), and low levels of CA9 in normal tissue (scale bars: 50 μm). Level adjustments were made to images in Adobe Photoshop post-acquisition for clarity (equal changes applied to the entire image). Related to Fig 2A–C.

Source data are available online for this figure.

 © 2018 The Authors

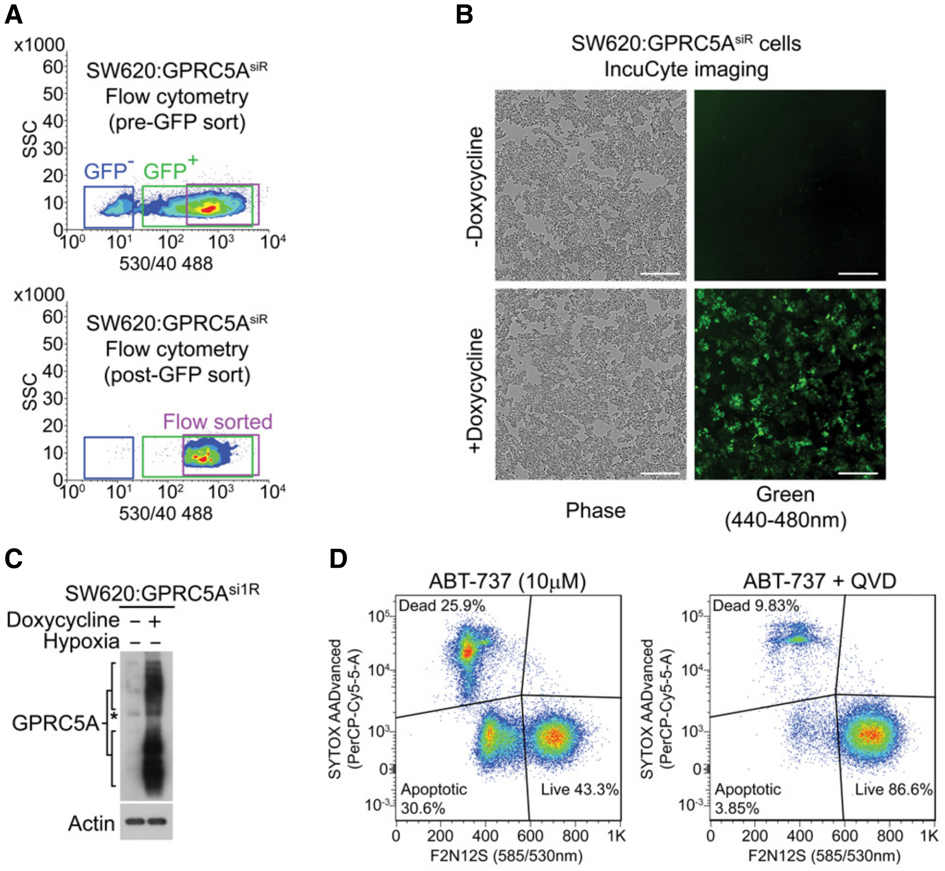

Figure EV3. Generation of doxycycline-inducible SW620:GPRC5A^{si1R} cells and optimisation of flow cytometry for use in violet ratiometric membrane asymmetry apoptosis assay.

A Lentivirally transduced and puromycin-selected SW620:GPRC5A^{si1R} cells were doxycycline-induced (2.5 μg/ml) for 48 h and flow-sorted (BD Influx) based on medium/high TurboGFP expression (note that pCW57-GFP-2A-GPRC5A^{si1R} uses the P2A self-cleaving peptide to produce separate TurboGFP and GPRC5A^{si1R} cDNA). Pre- and post-flow-sorted profiles are shown, confirming near 100% expression. Related to Figs 3D, and 4H, I, and L.

B Confirmation of TurboGFP expression in doxycycline (2.5 μg/ml)-treated SW620:GPRC5A^{si1R} cells. Phase contrast and green (TurboGFP) images were obtained using the IncuCyte ZOOM live cell imaging system (scale bars: 300 μm). Level adjustments were made to images in Adobe Photoshop post-acquisition for clarity (equal changes applied to the entire image). Related to Figs 3D, and 4H, I, and L.

C Western blotting confirms GPRC5A overexpression in doxycycline-treated SW620:GPRC5A^{si1R} cells. Cells were treated for 72 h with doxycycline (2.5 μg/ml) in normoxia prior to harvest. Related to Figs 3D, and 4H, I, and L. Asterisk (*) indicates non-specific band.

D Optimisation of the violet ratiometric membrane asymmetry apoptosis assay for flow cytometry. SW620 cells were treated for 24 h with the pan-BCL2 family inhibitor ABT-737 (10 μM). Live, dead and apoptotic cells were gated using FlowJo (v10). A representative example is shown. Related to Fig 3E.

Source data are available online for this figure.

© 2018 The Authors

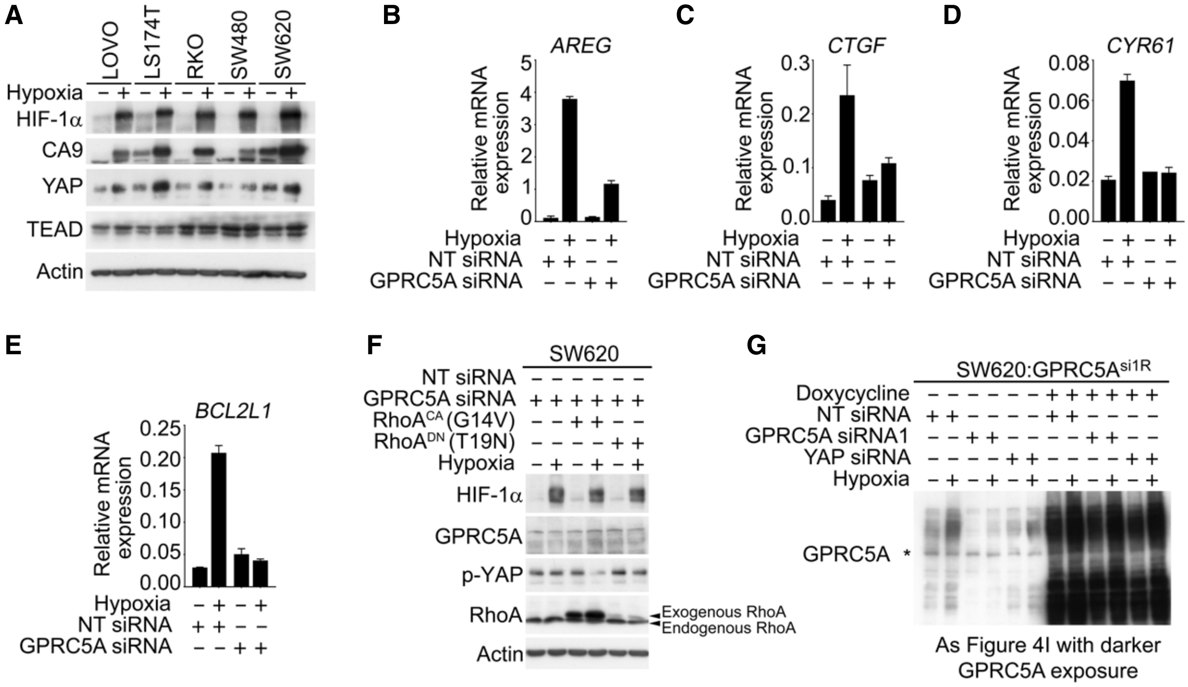

Figure EV4. GPRC5A promotes hypoxic cell survival via YAP.

A Hypoxia induced increased total YAP expression in a panel of colorectal cancer cell lines. Actin confirmed equal loading; blots are representative of at least two independent experiments. Related to Fig 4.

B–E qRT–PCR analysis revealed that hypoxia increased the expression of known YAP target genes *AREG*, *BCL2L1* (BCL-XL), *CTGF* and *CYR61*. Representative experiments are shown. Representative examples of $n = 3$ independent experiments are shown; data are presented as mean ± SD.

F Increased expression of YAP Ser397 in GPRC5A-depleted cells was overridden by expression of constitutively active RhoA (G14V) in hypoxia. Expression of dominant negative RhoA (T19N) did not further increase YAP Ser397 phosphorylation in response to GPRC5A depletion.

G Darker exposure of the GPRC5A blot in Fig 4I, confirming expression of 30–40 kDa and 80 kDa species. Note that YAP siRNA partially diminishes GPRC5A in line with the existence of positive HIF-GPRC5A-YAP feedback loop. Asterisk (*) indicates non-specific band.

Source data are available online for this figure.

 © 2018 The Authors

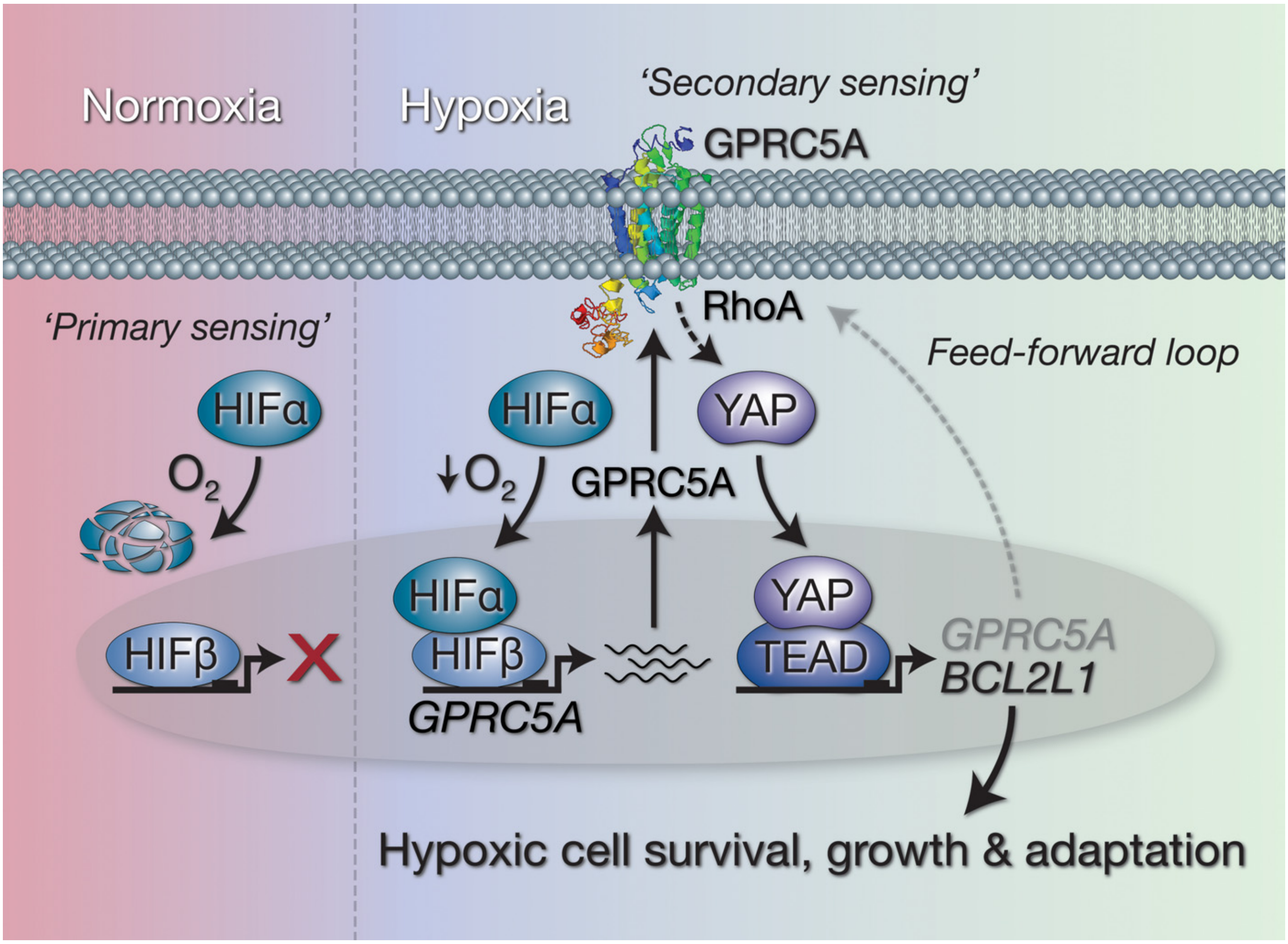

Figure EV5. Hypoxic cancer cell survival involves a HIF-GPRC5A-YAP axis.

Working model of the HIF-GPRC5A-YAP axis described in this study. Under normoxic conditions, "primary metabolic sensors" HIF-α subunits are degraded via VHL-dependent proteolysis meaning basal *GPRC5A* expression levels are low. When cells experience reduced O_2 levels (hypoxia), HIF-1α and HIF-2α subunits are stabilised and move to the nucleus where they interact with HIF-1β/ARNT to drive the expression of *GPRC5A*. GPRC5A acts as a "secondary metabolic sensor" (potentially sensing microenvironmental conditions, nutrients and/or metabolites) in hypoxia, and as its expression increases at the plasma membrane, YAP is stabilised via RhoA to induce expression of anti-apoptotic *BCL2L1* (encoding BCL-XL). In addition, activation of YAP further increases GPRC5A expression leading to a feed-forward "oncogenic loop" that promotes cellular adaptation and survival under low oxygen conditions.

© 2018 The Authors
