# Supplementary material for: Cancer cell adaptation to hypoxia involves a HIF‐GPRC5A‐YAP axis
Source: EMBO Mol Med. 2018 Aug 24;10(11):e8699. doi: 10.15252/emmm.201708699 (PMC6220329; doi:10.15252/emmm.201708699)

Figure EV3 source data

Unprocessed images for the indicated figures are shown. Boxes are used to indicate the exposure and/or area used in the paper where ambiguous.

Figure EV3 B

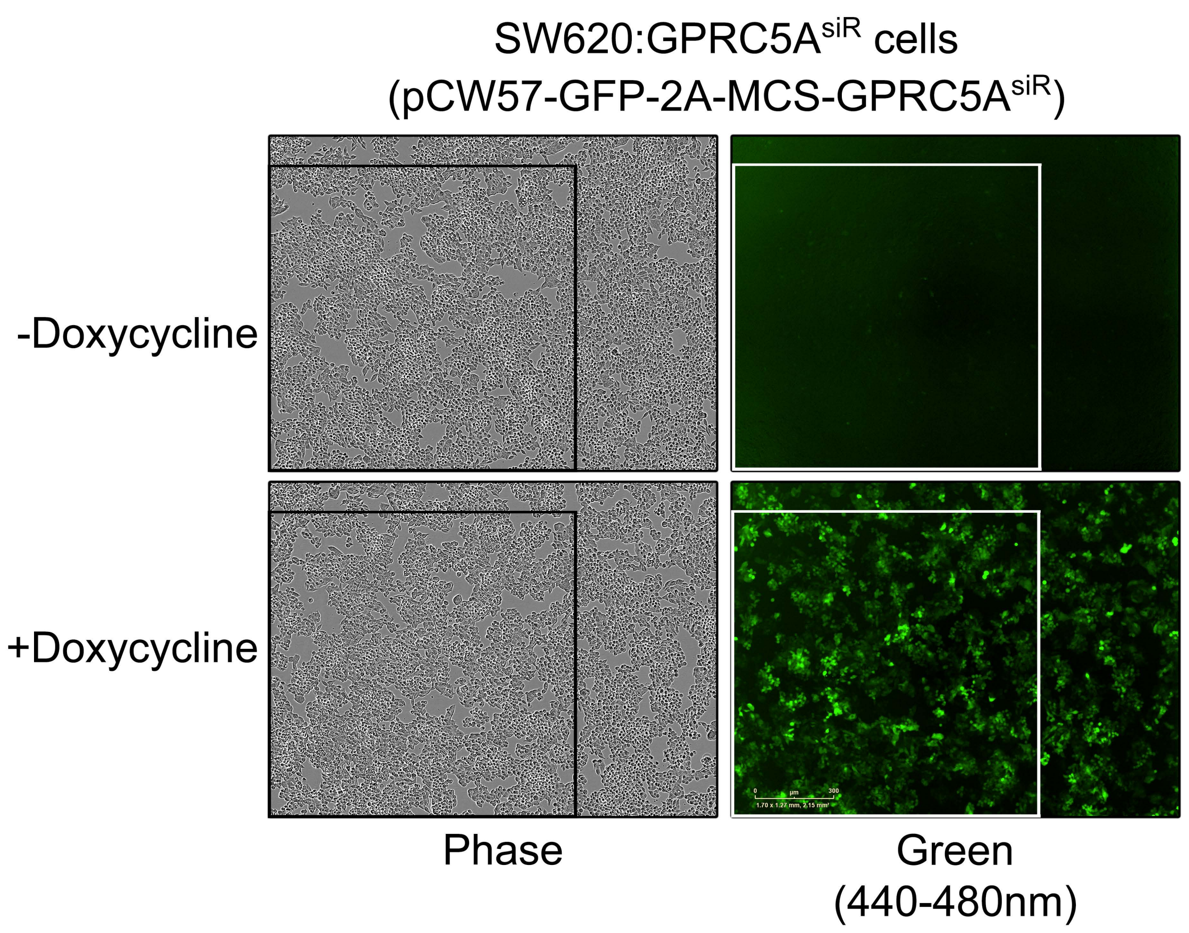

Figure EV3 C

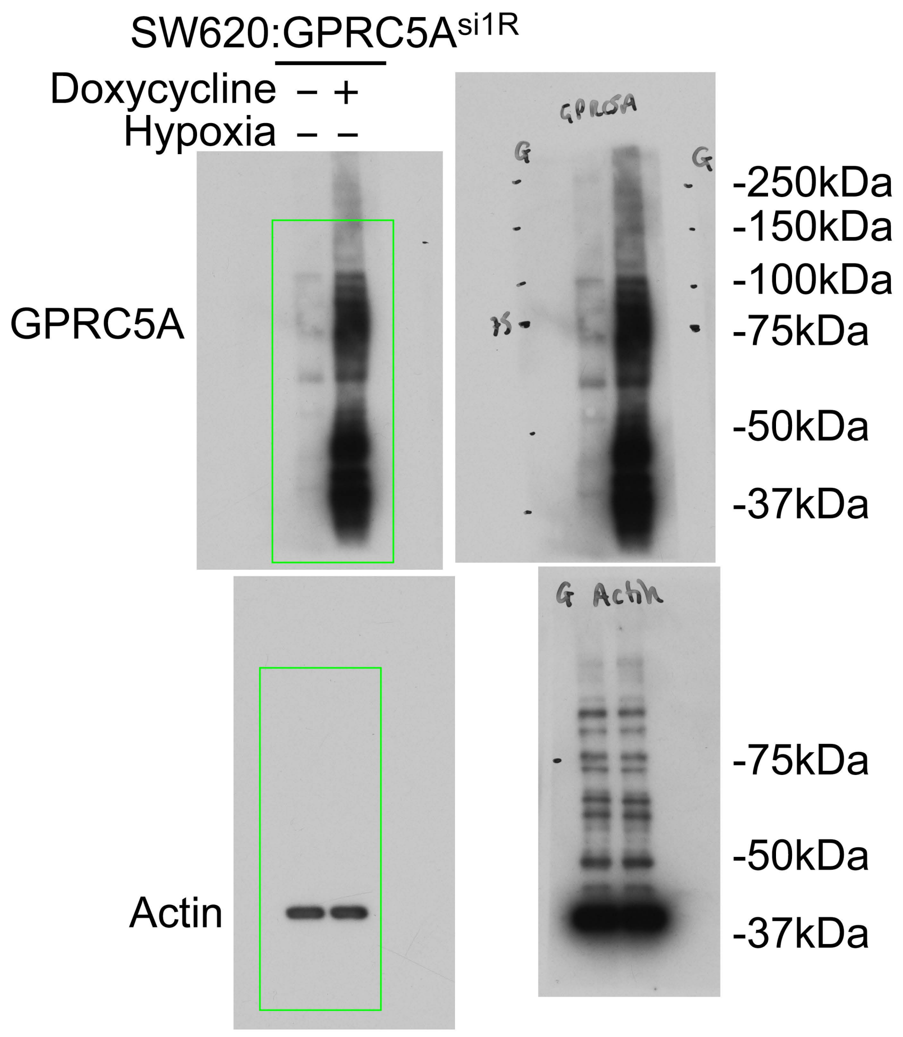

Supplement: Supplementary file 3 — Source Data for Expanded View [file EMMM-10-e8699-s007.zip › EV_figure_source_data/FigEV3_source_data_V3.pdf]
