# Supplementary figures and images for: Cancer cell adaptation to hypoxia involves a HIF‐GPRC5A‐YAP axis
Source: EMBO Mol Med. 2018 Aug 24;10(11):e8699. doi: 10.15252/emmm.201708699 (PMC6220329; doi:10.15252/emmm.201708699)

# Figure EV2 source data

Unprocessed images for the indicated figures are shown.

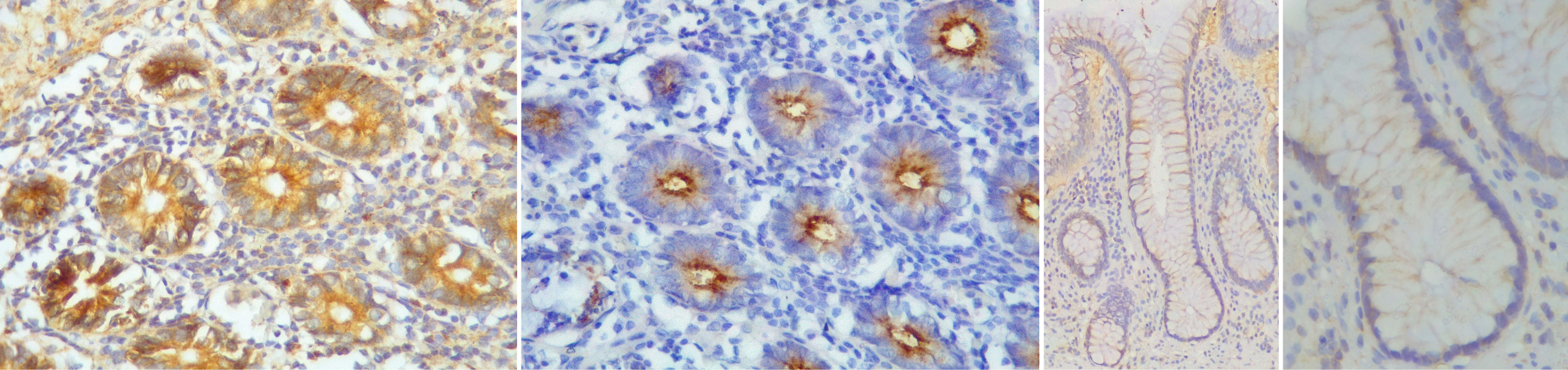

Supplement: Supplementary file 3 — Source Data for Expanded View [file EMMM-10-e8699-s007.zip › EV_figure_source_data/FigEV2_source_data_V3.pdf]

Figure 2 source data

Figure 2A

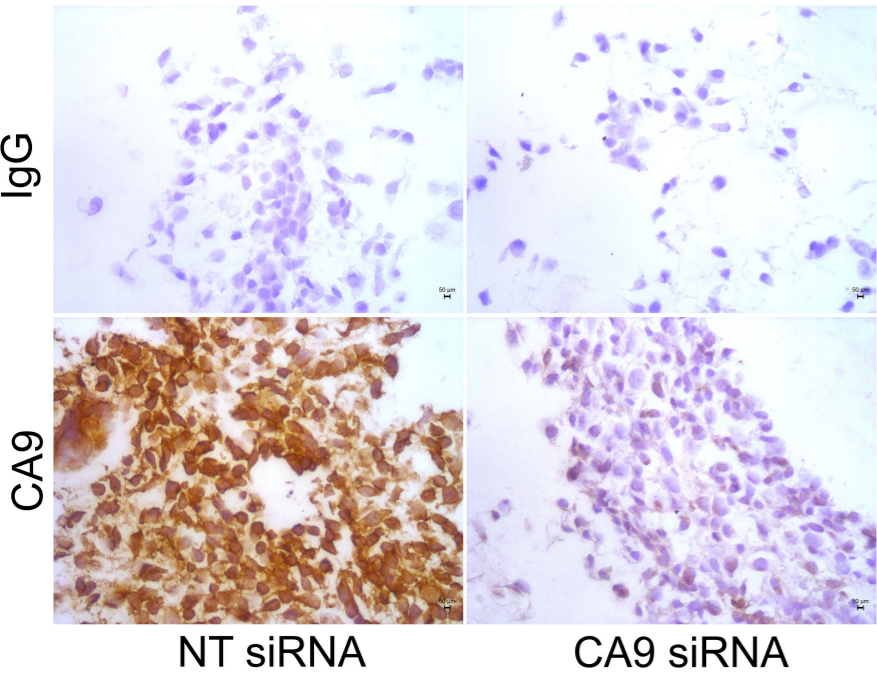

Figure 2B

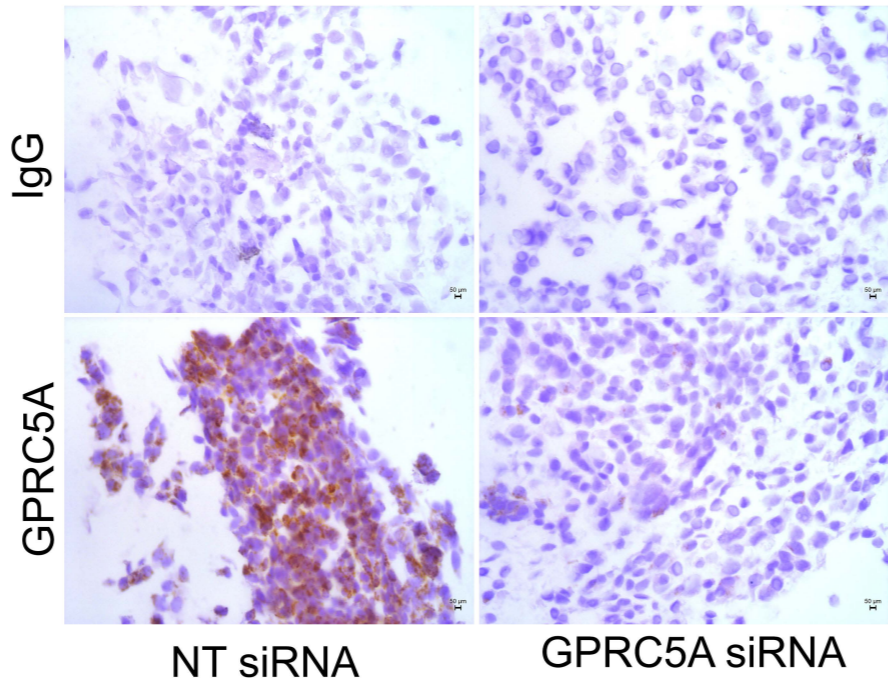

Figure 2C

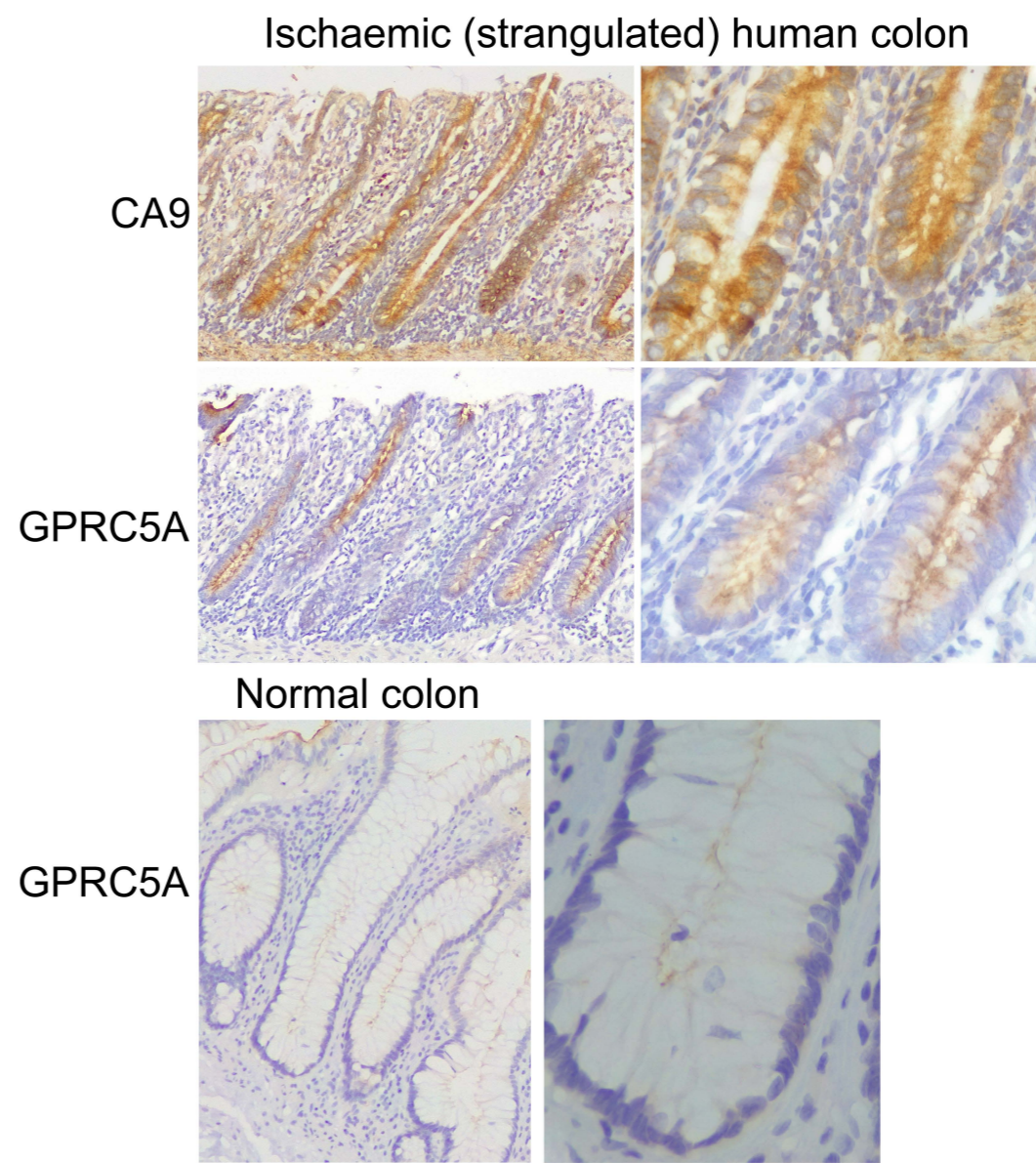

Figure 2E

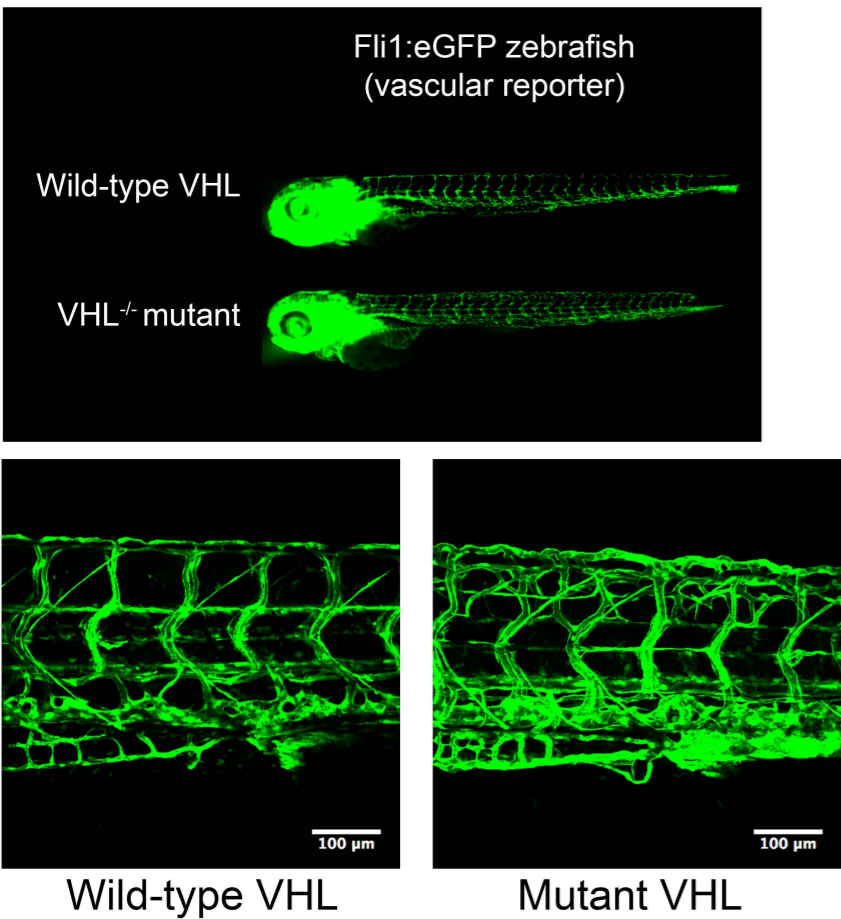

Figure 2F

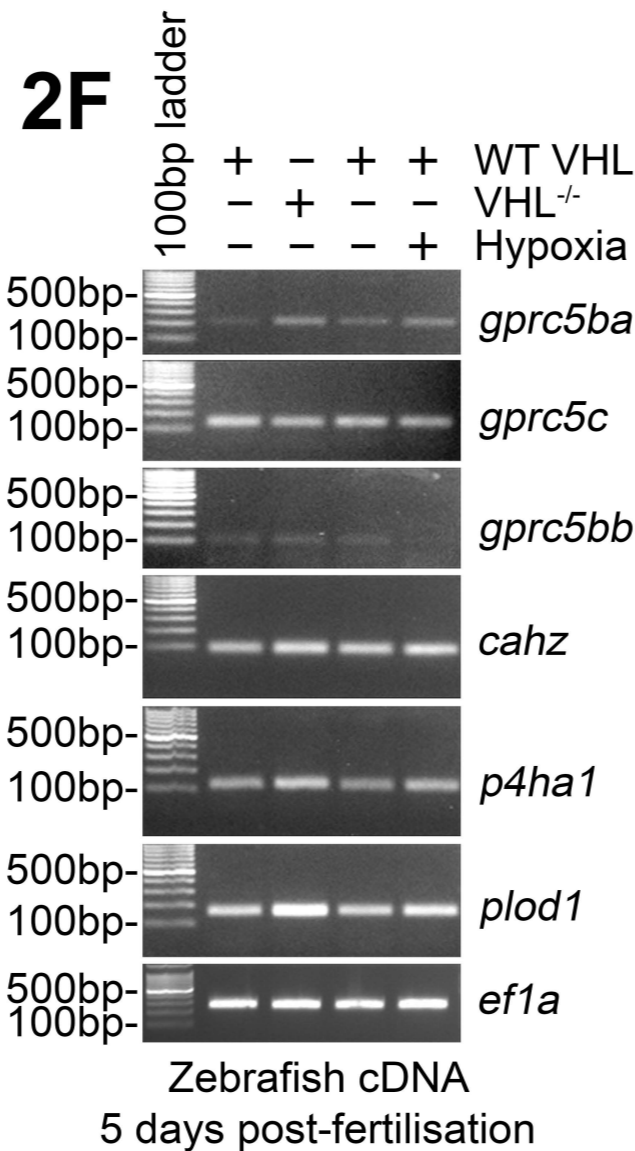

Supplement: Supplementary file 6 — Source Data for Figure 2 [file EMMM-10-e8699-s004.pdf]
