# Supplementary material for: Cancer cell adaptation to hypoxia involves a HIF‐GPRC5A‐YAP axis
Source: EMBO Mol Med. 2018 Aug 24;10(11):e8699. doi: 10.15252/emmm.201708699 (PMC6220329; doi:10.15252/emmm.201708699)

Figure 1 source data

Unprocessed blots for the indicated figures are shown. Green boxes are used to indicate the exposure and/or area used in the paper where ambiguous.

Figure 1B

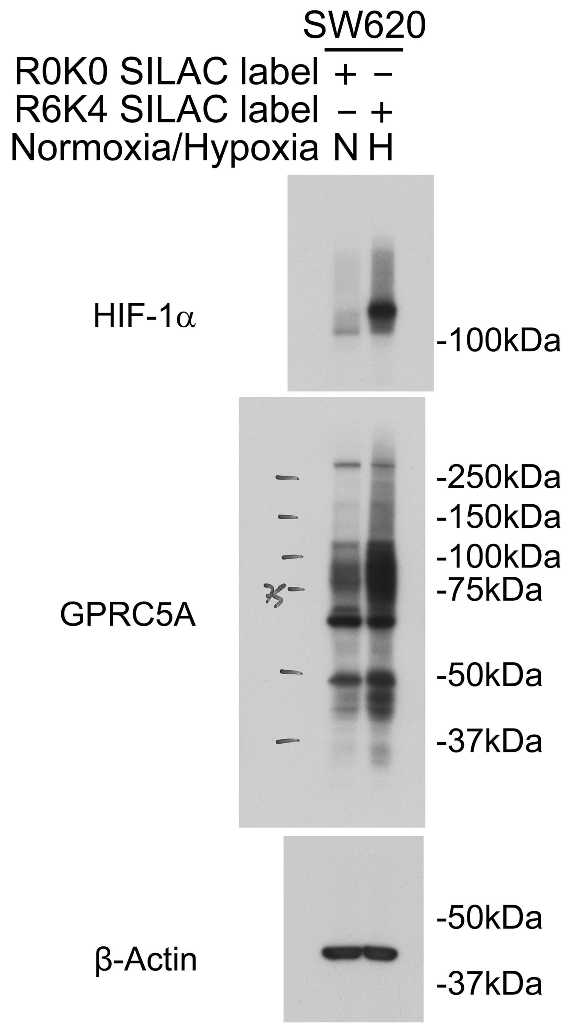

Figure 1C

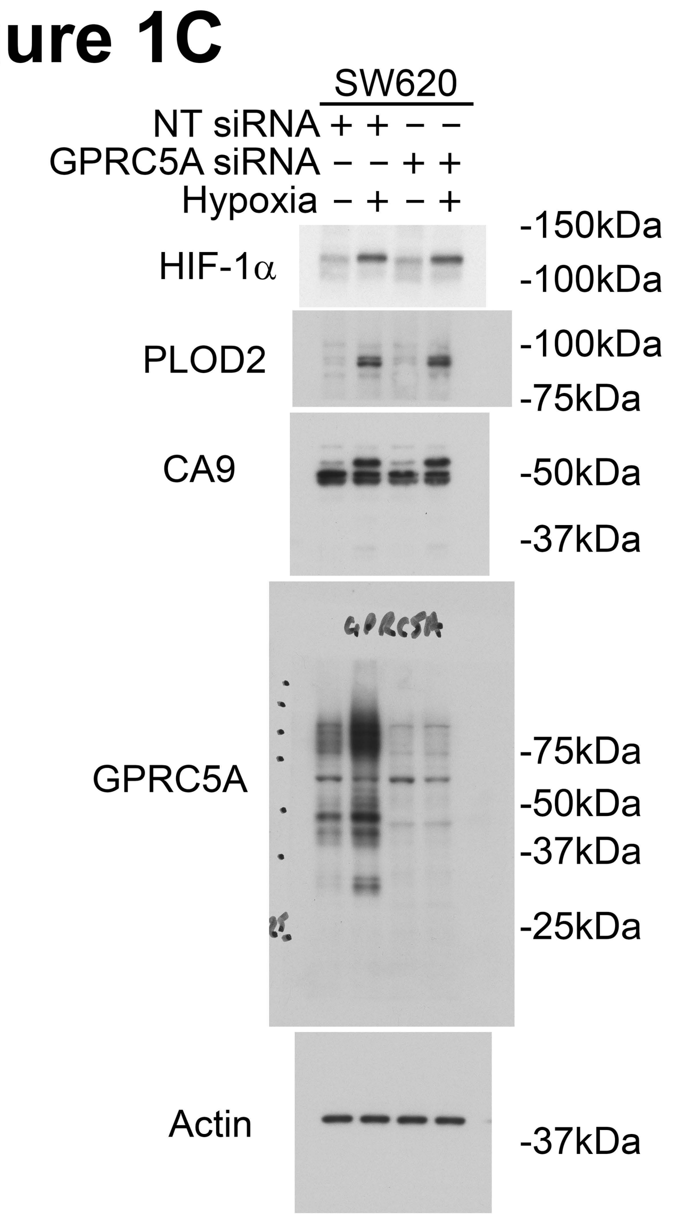

Figure 1D

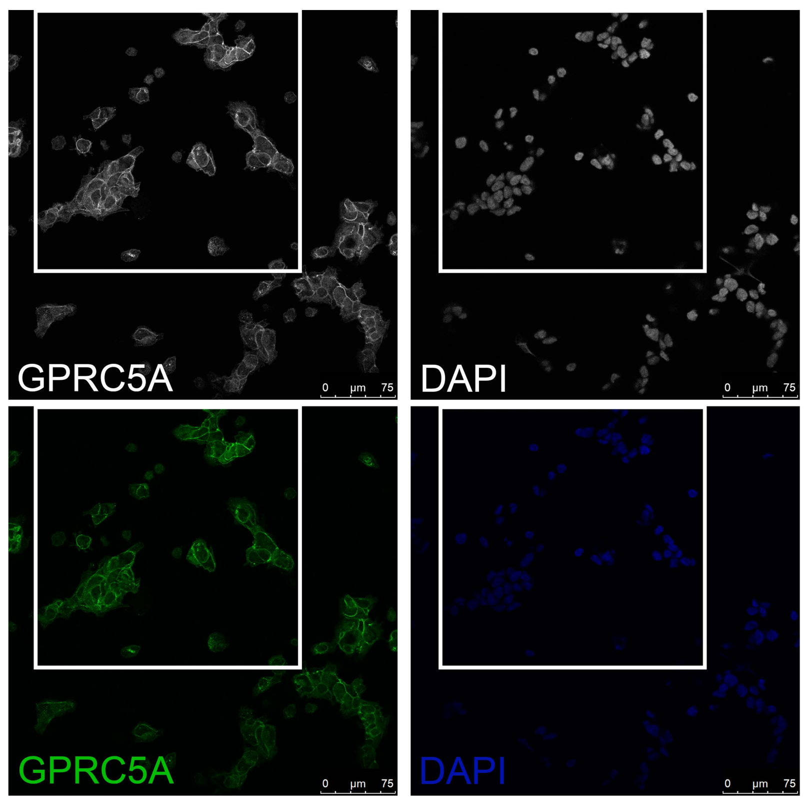

Figure 1E

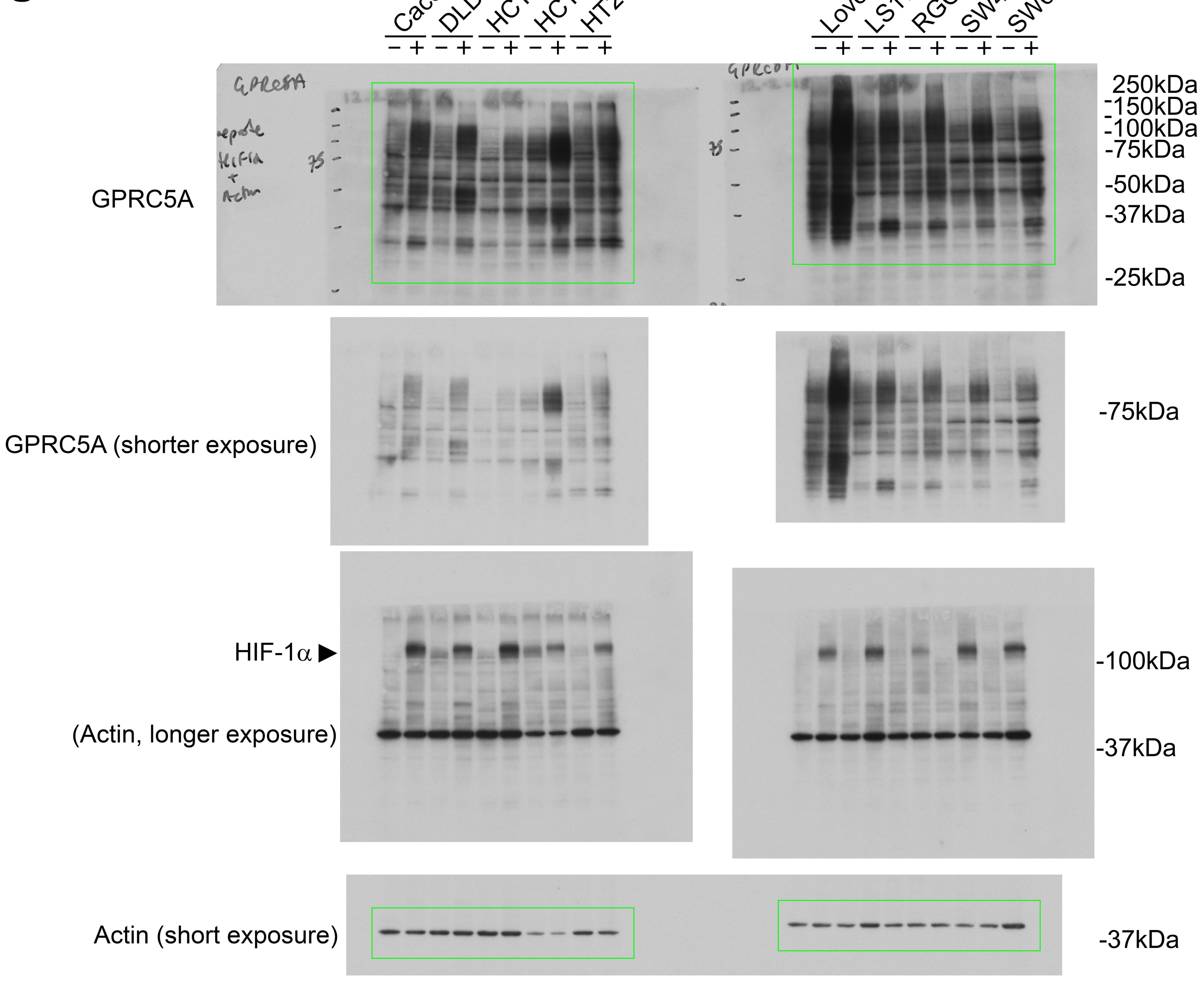

Figure 1F

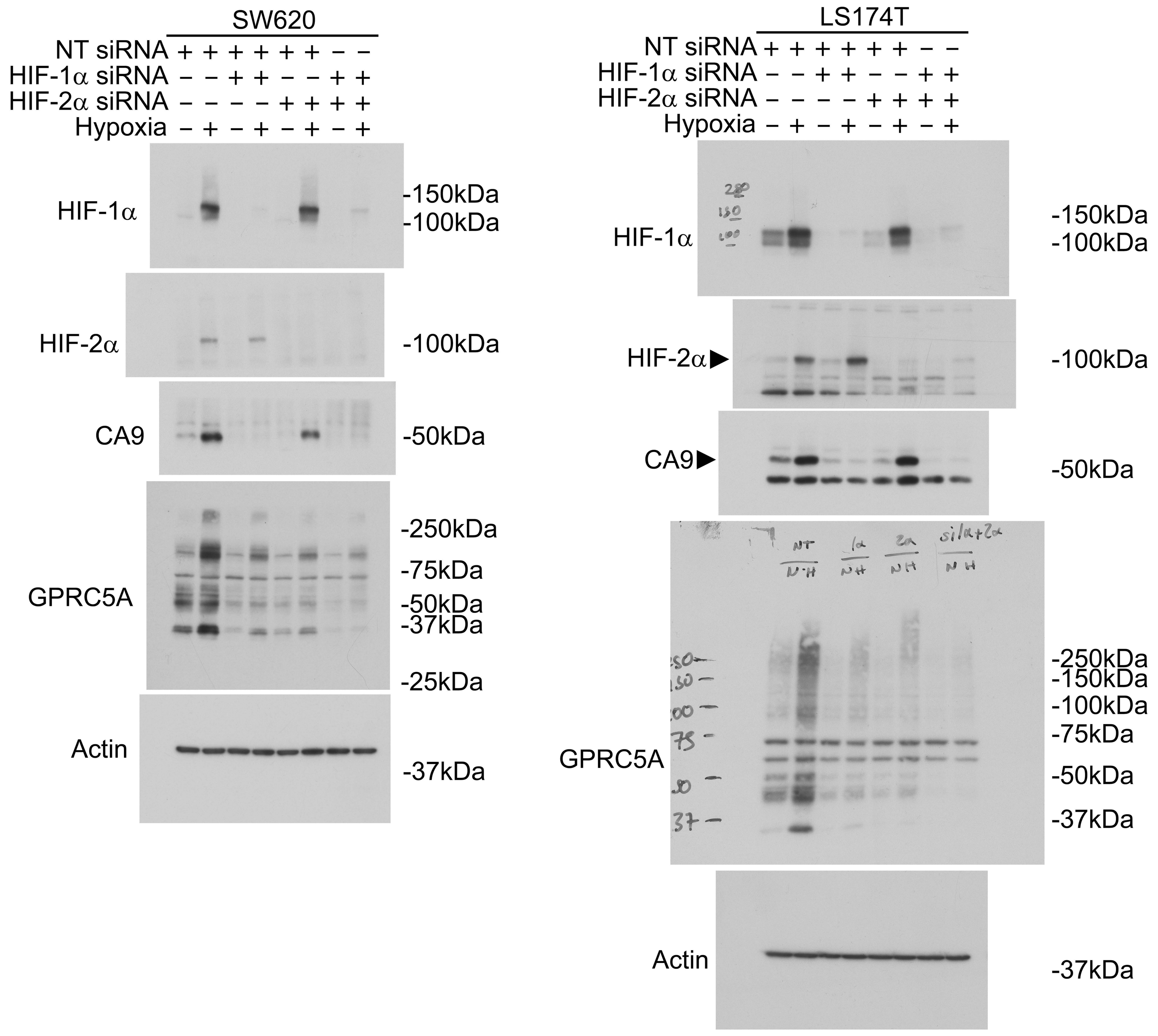

Figure 1G

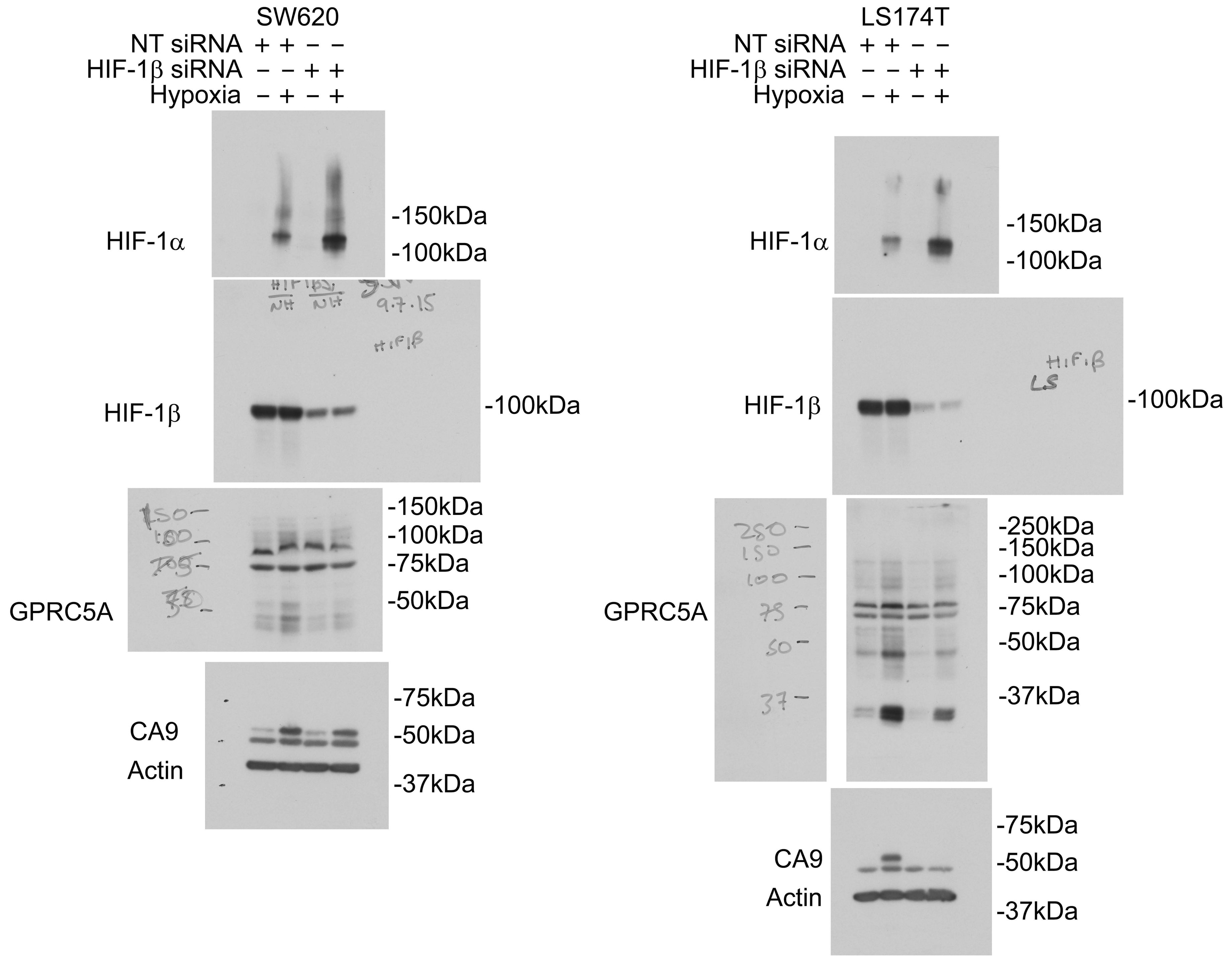

Figure 1H

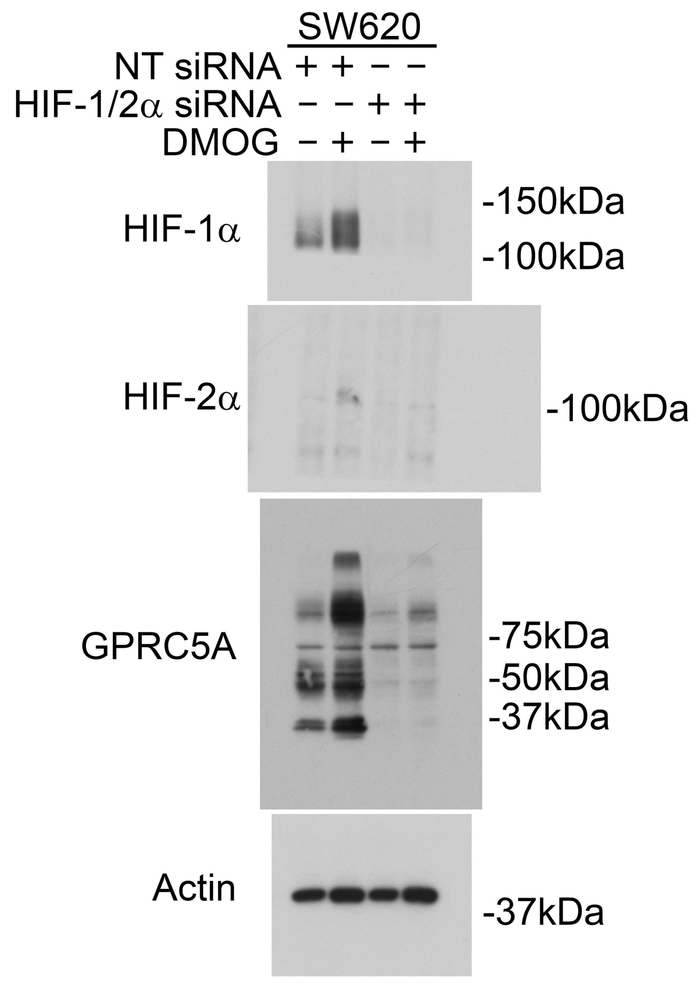

Supplement: Supplementary file 5 — Source Data for Figure 1 [file EMMM-10-e8699-s003.pdf]
