# Supplementary material for: Cancer cell adaptation to hypoxia involves a HIF‐GPRC5A‐YAP axis
Source: EMBO Mol Med. 2018 Aug 24;10(11):e8699. doi: 10.15252/emmm.201708699 (PMC6220329; doi:10.15252/emmm.201708699)

Figure 3 source data

Unprocessed blots for the indicated figures are shown. Green boxes are used to indicate the exposure and/or area used in the paper where ambiguous.

Figure 3A

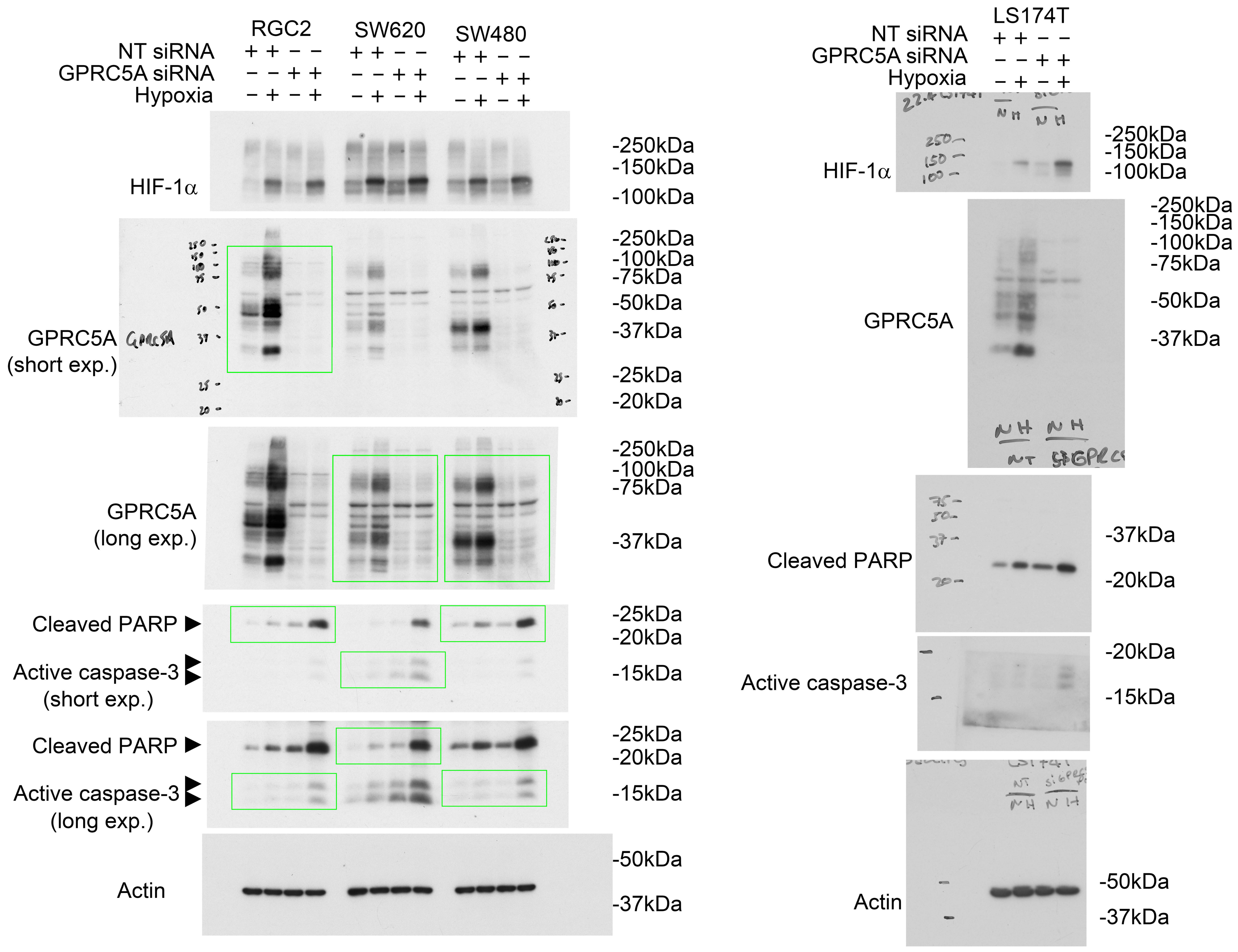

Figure 3B

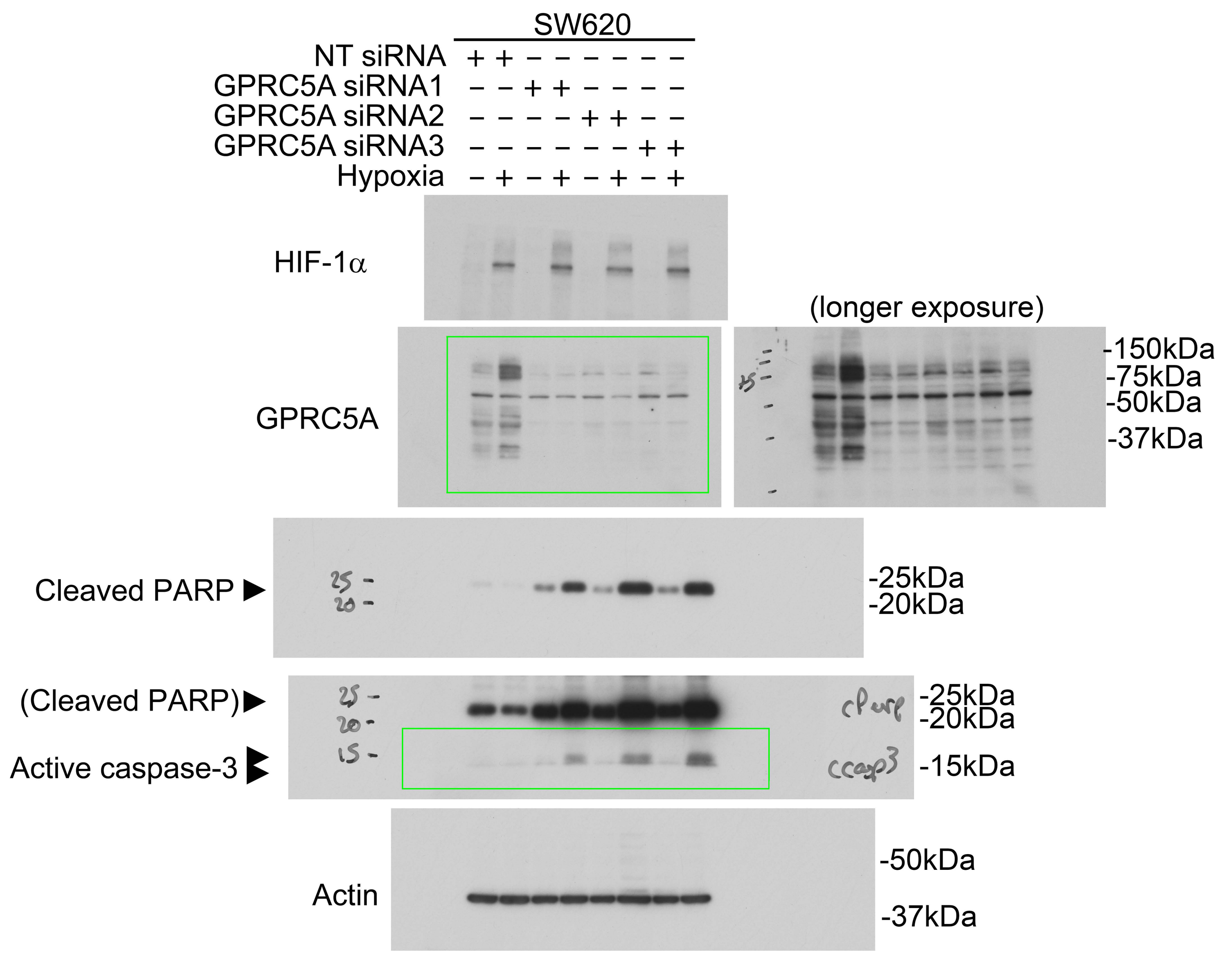

Figure 3D

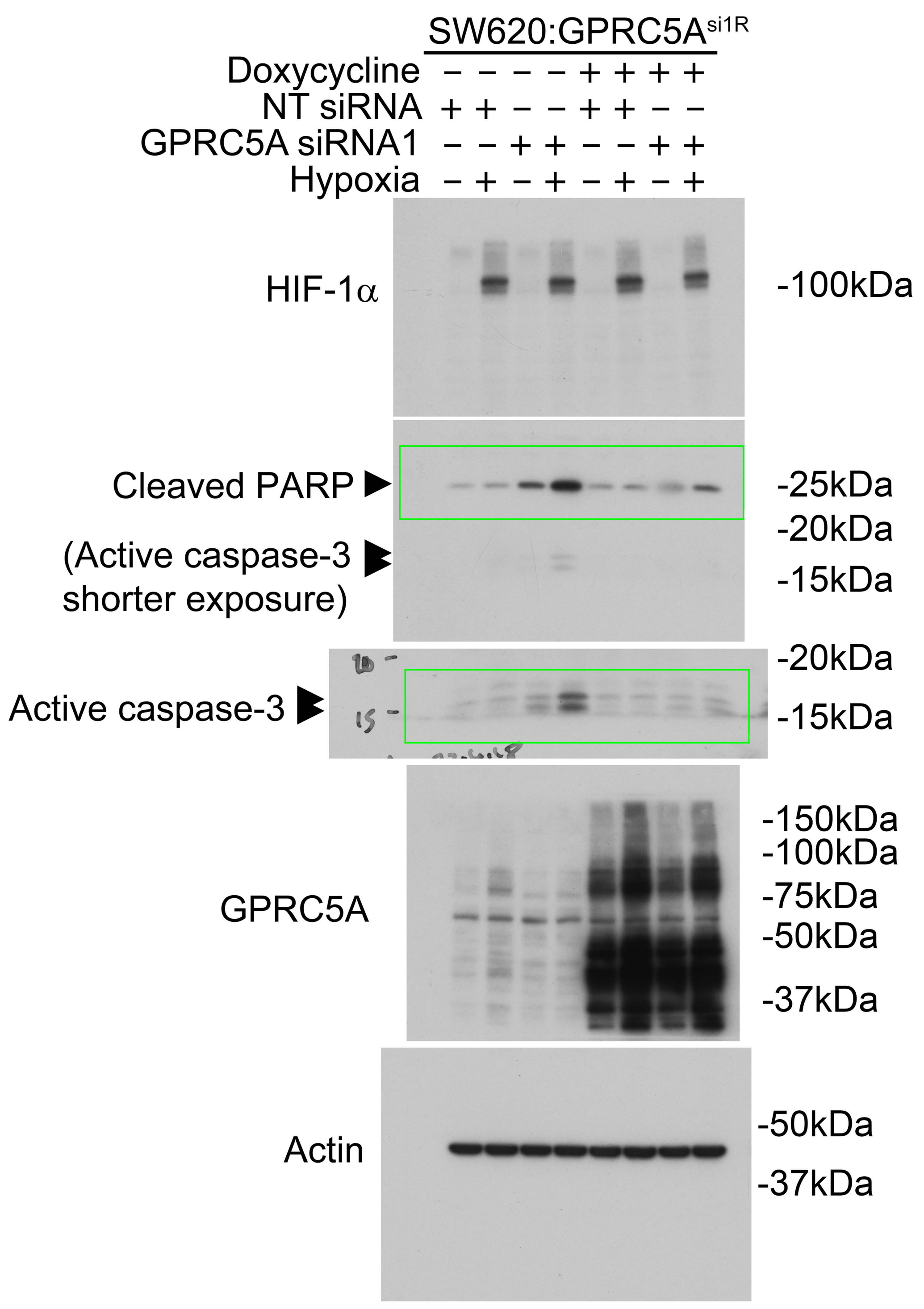

Figure 3E

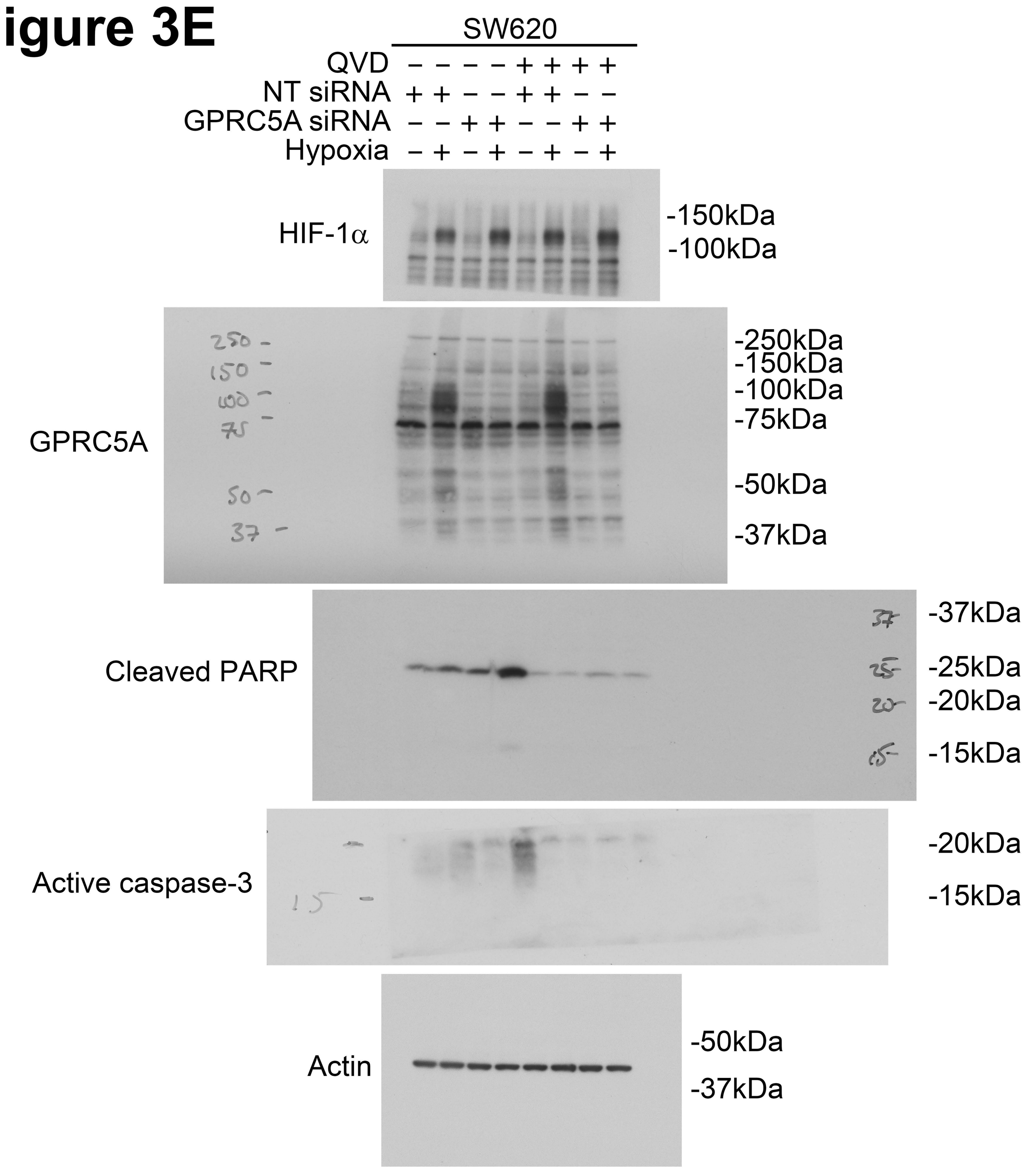

Supplement: Supplementary file 7 — Source Data for Figure 3 [file EMMM-10-e8699-s005.pdf]
