# Supplementary material for: Cancer cell adaptation to hypoxia involves a HIF‐GPRC5A‐YAP axis
Source: EMBO Mol Med. 2018 Aug 24;10(11):e8699. doi: 10.15252/emmm.201708699 (PMC6220329; doi:10.15252/emmm.201708699)

Figure 4 source data

Unprocessed blots for the indicated figures are shown. Green boxes are used to indicate the exposure and/or area used in the paper where ambiguous.

Figure 4A

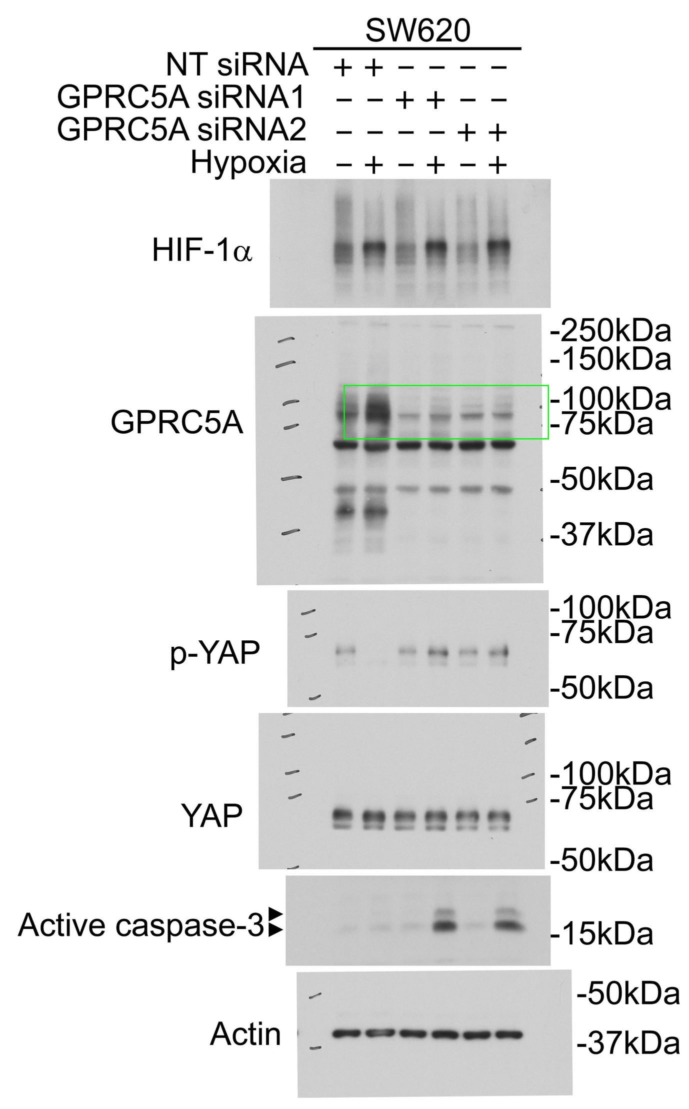

Figure 4B

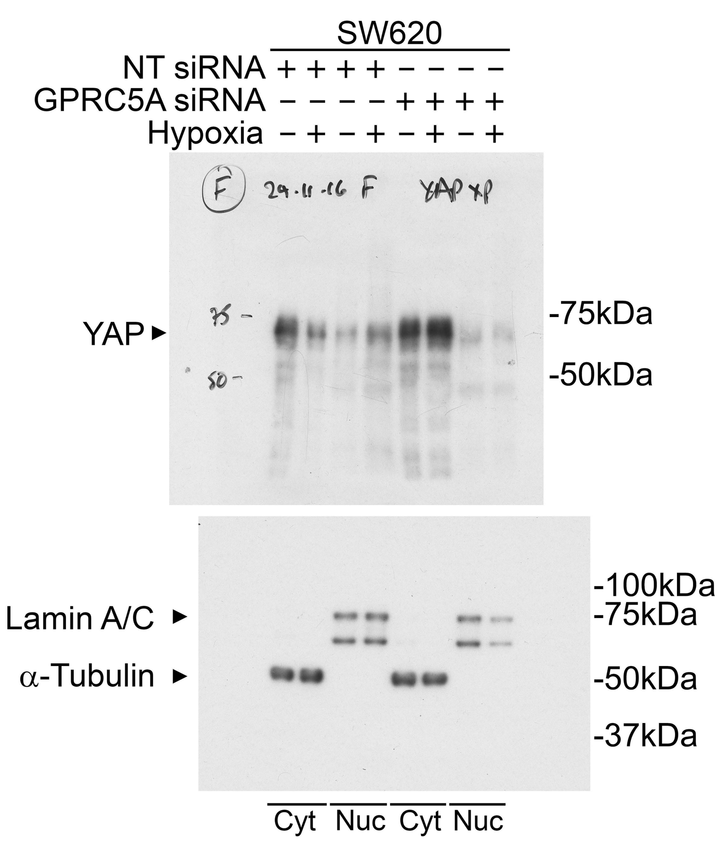

Figure 4D

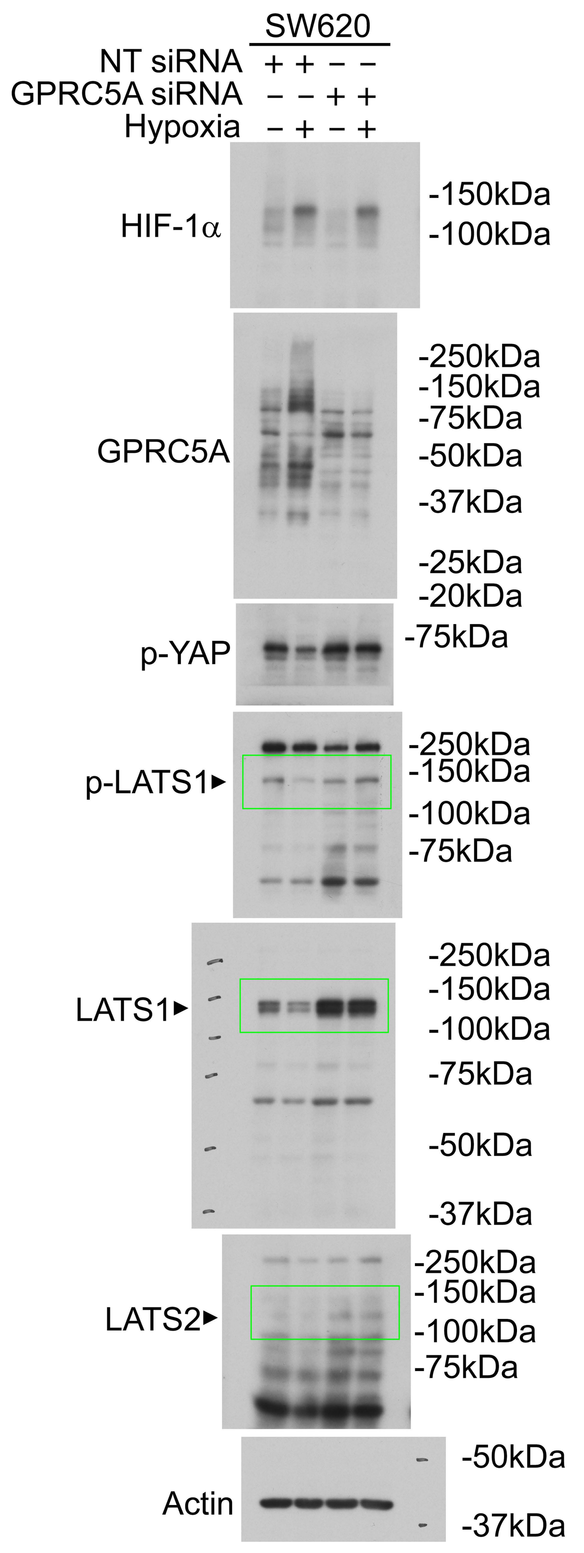

Figure 4E

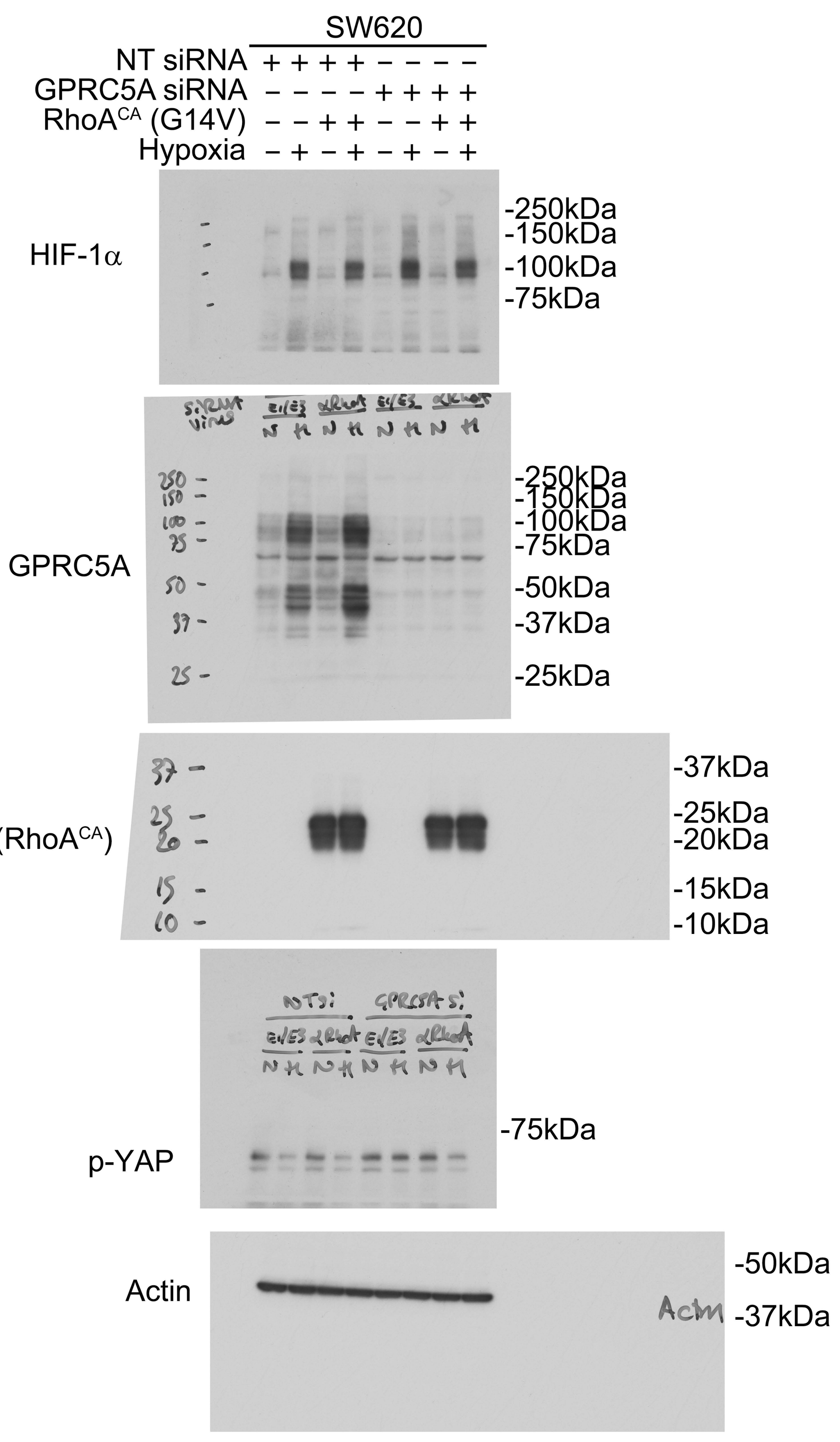

Figure 4G

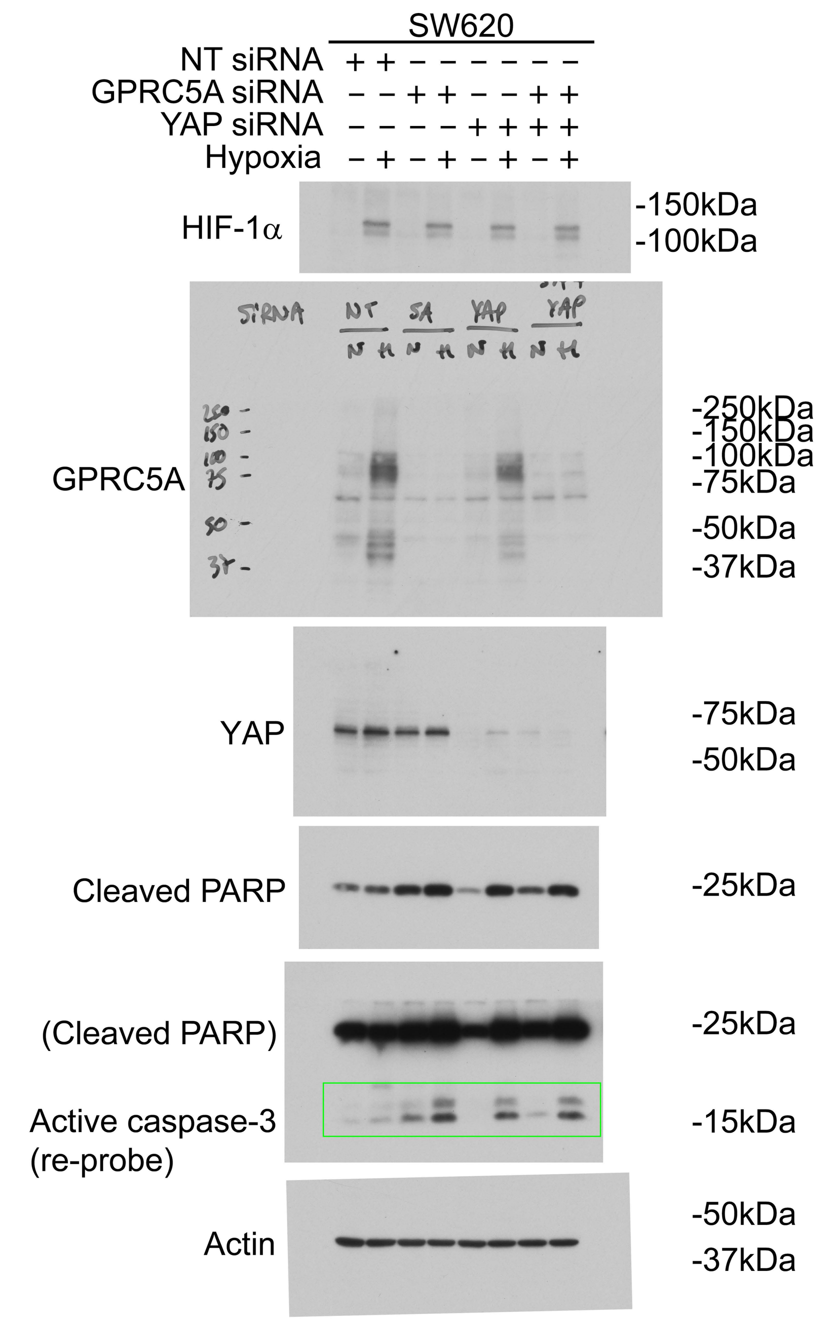

Figure 4I

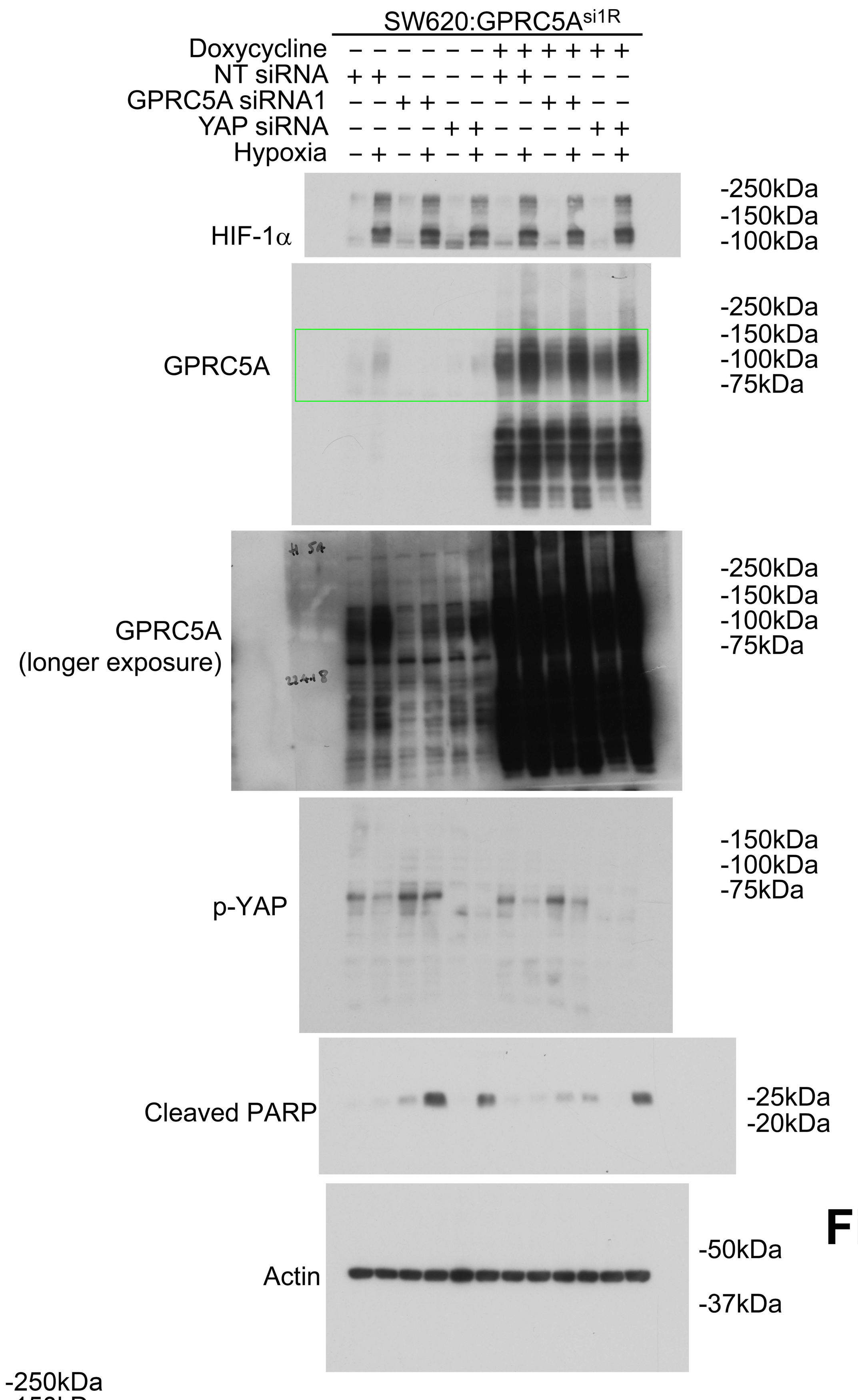

Figure 4J

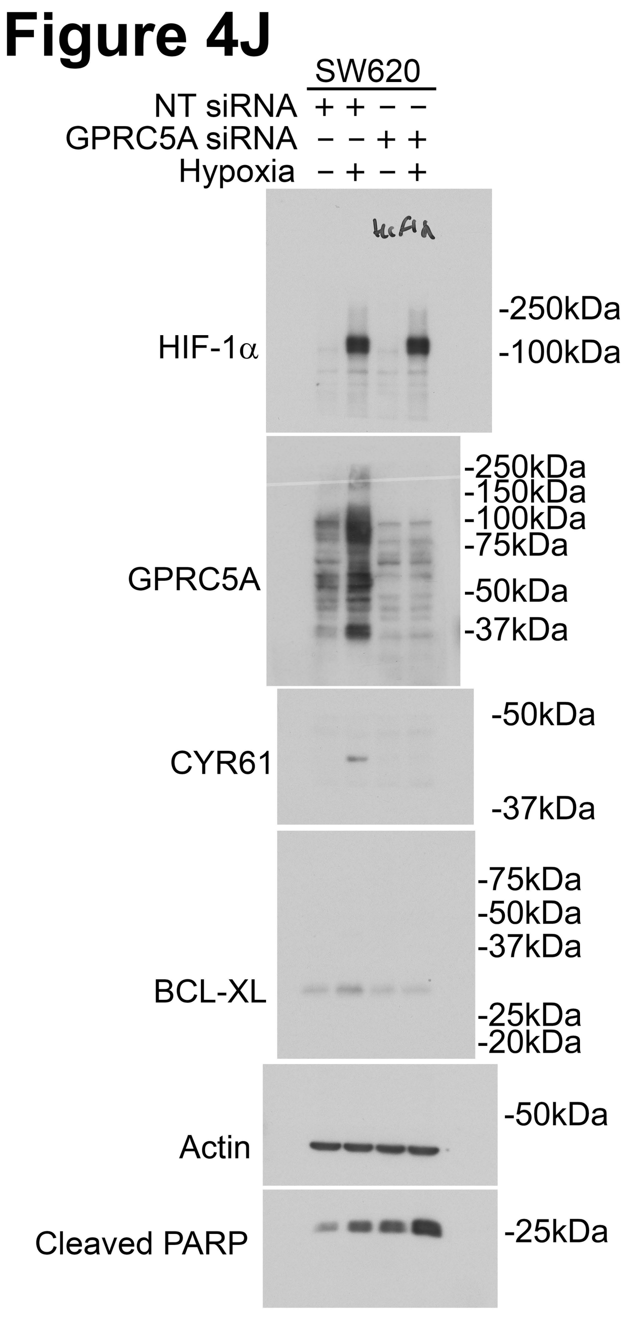

Figure 4K

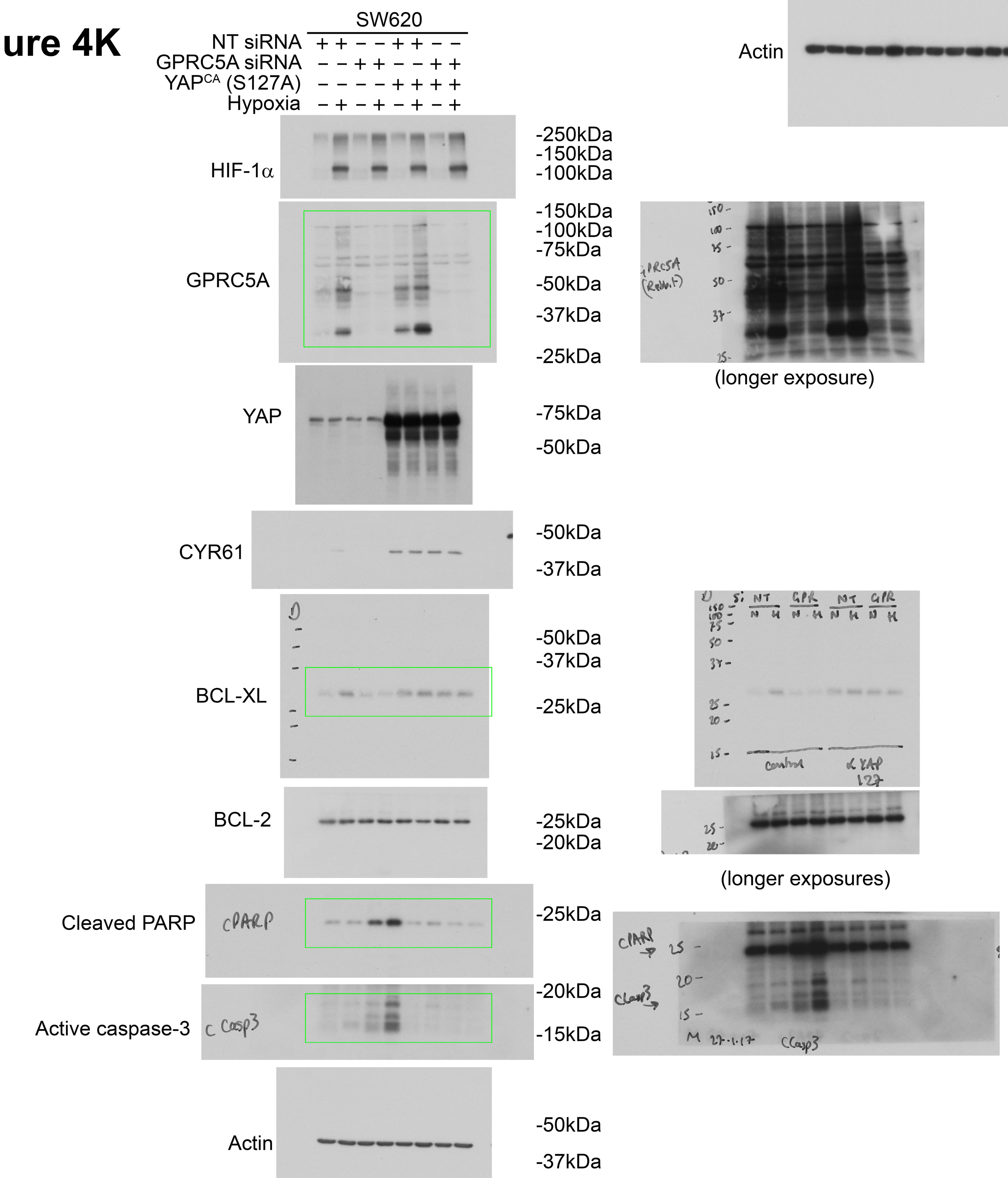

Figure 4L

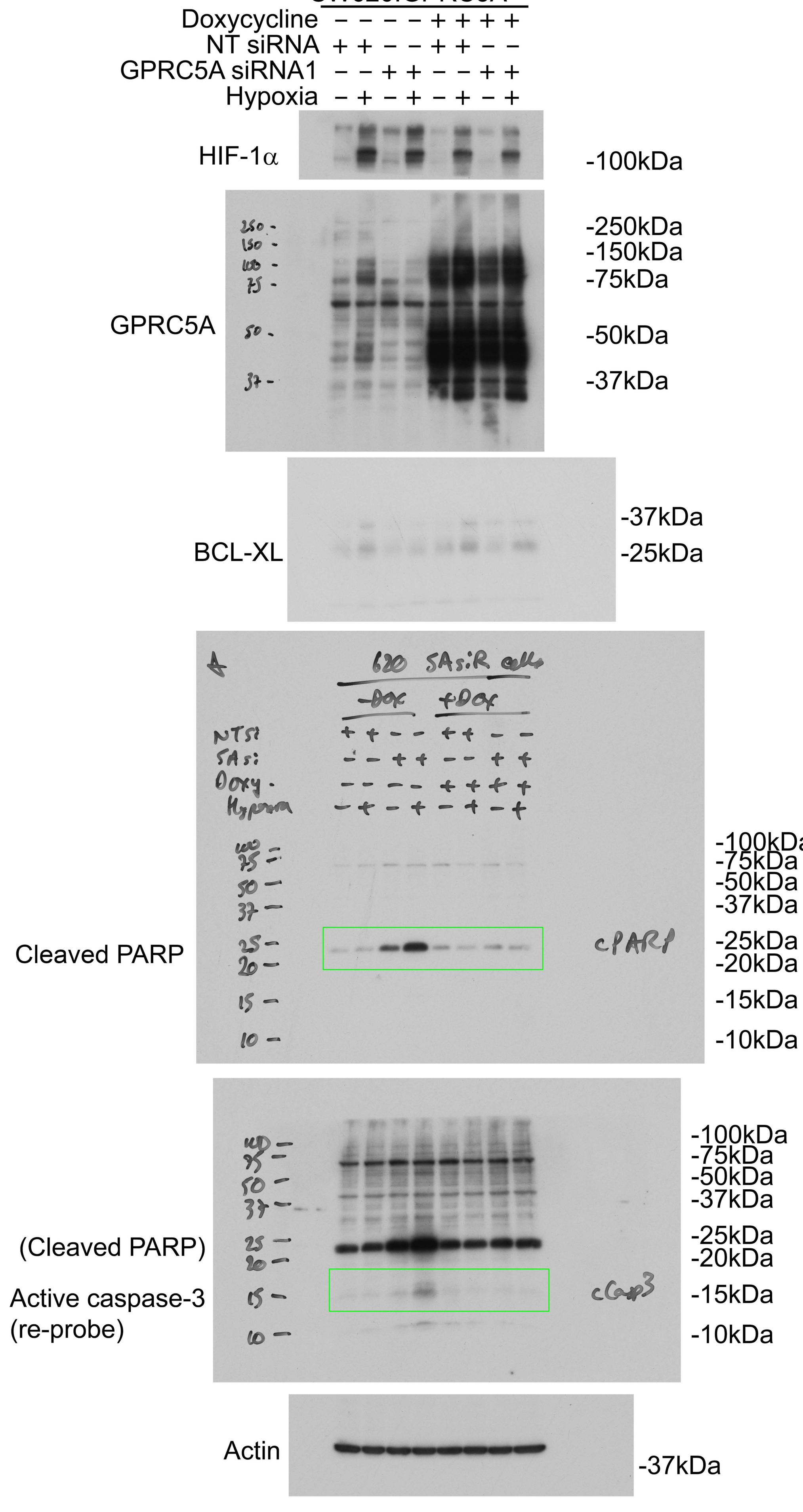

Supplement: Supplementary file 8 — Source Data for Figure 4 [file EMMM-10-e8699-s006.pdf]
